# Targeting BCL9/BCL9L enhances antigen presentation by promoting conventional type 1 dendritic cell (cDC1) activation and tumor infiltration

**DOI:** 10.1038/s41392-024-01838-9

**Published:** 2024-05-29

**Authors:** Fenglian He, Zhongen Wu, Chenglong Liu, Yuanyuan Zhu, Yan Zhou, Enming Tian, Rina Rosin-Arbesfeld, Dehua Yang, Ming-Wei Wang, Di Zhu

**Affiliations:** 1 Department of Pharmacology, Minhang Hospital, and Key Laboratory of Smart Drug Delivery, Shanghai Engineering Research Center of Immune Therapy, School of Pharmacy, Fudan University, Shanghai, 201203 China; 2 The National Center for Drug Screening and the CAS Key Laboratory of Receptor Research, Shanghai Institute of Materia Medica, Chinese Academy of Sciences (CAS), Shanghai, 201203 China; 3 Department of Microbiology and Immunology, Sackler Faculty of Medicine, Tel Aviv University, Tel Aviv, Israel; 4 Department of Pharmacology, School of Basic Medical Sciences, Fudan University, Shanghai, 200032 China; 5 Research Center for Deepsea Bioresources, Sanya, China; 6 Department of Chemistry, School of Science, The University of Tokyo, Tokyo, Japan; 7 Engineering Research Center of Tropical Medicine Innovation and Transformation of Ministry of Education, School of Pharmacy, Hainan Medical University, Haikou, China; 8 Shandong Academy of Pharmaceutical Science, Jinan, China

**Keywords:** Cancer microenvironment, Tumour immunology, Tumour immunology

## Abstract

Conventional type 1 dendritic cells (cDC1) are the essential antigen-presenting DC subset in antitumor immunity. Suppressing B-cell lymphoma 9 and B-cell lymphoma 9-like (BCL9/BCL9L) inhibits tumor growth and boosts immune responses against cancer. However, whether oncogenic BCL9/BCL9L impairs antigen presentation in tumors is still not completely understood. Here, we show that targeting BCL9/BCL9L enhanced antigen presentation by stimulating cDC1 activation and infiltration into tumor. Pharmacological inhibition of BCL9/BCL9L with a novel inhibitor hsBCL9_z96_ or *Bcl9/Bcl9l* knockout mice markedly delayed tumor growth and promoted antitumor CD8^+^ T cell responses. Mechanistically, targeting BCL9/BCL9L promoted antigen presentation in tumors. This is due to the increase of cDC1 activation and tumor infiltration by the XCL1-XCR1 axis. Importantly, using single-cell transcriptomics analysis, we found that *Bcl9/Bcl9l* deficient cDC1 were superior to wild-type (WT) cDC1 at activation and antigen presentation via NF-κB/IRF1 signaling. Together, we demonstrate that targeting BCL9/BCL9L plays a crucial role in cDC1-modulated antigen presentation of tumor-derived antigens, as well as CD8^+^ T cell activation and tumor infiltration. Targeting BCL9/BCL9L to regulate cDC1 function and directly orchestrate a positive feedback loop necessary for optimal antitumor immunity could serve as a potential strategy to counter immune suppression and enhance cancer immunotherapy.

## Introduction

Antigen presentation is indispensable to antitumor responses. In order to elicit effective antitumor CD8^+^ T cell responses, antigen presentation must be successful in two major events: First, tumor antigens are captured by antigen-presenting cells (APCs), processed into peptide fragments and presented on APCs with major histocompatibility complex class I (MHC-I) or human leukocyte antigen class I (HLA-I) to prime CD8^+^ T cells. Second, the activated CD8^+^ T cells recognize the tumor antigens that directly presented by APCs and then kill the tumor cells.^1^ Admittedly, conventional type 1 dendritic cells (cDC1) are considered the superior DC subset to present tumor antigens onto MHC-I to prime CD8^+^ T cells.^2,3^ Previous studies revealed that BATF3^−/−^ mice that selectively lack cDC1 demonstrate deficient cross-presentation and impair antitumor immunity.^4–6^ In human cancers, increasing evidence indicate that intratumoral cDC1 are associated with the improved prognosis and responses to cancer immunotherapy.^7–9^ However, DCs are often dysfunctional, immature or even immunosuppressive, absent antigen-presenting capability for cross-priming within the tumor microenvironment (TME). Moreover, cDC1 are rare because TME excludes cDC1 from tumors through various mechanisms.^10^ Thus, approaches aimed to enhance antigen presentation, including increasing cDC1 activation and tumor infiltration may therefore promote antitumor immunity and improve cancer immunotherapy.

The Wnt/β-catenin signaling is not only critical in the regulation of tumor carcinogenesis, but also favors immune invasion in many malignancies.^11,12^ An increasing number of studies have reported that Wnt/β-catenin signaling pathway regulates DC functions.^13^ Loss of β-catenin in CD11c^+^ DCs during priming attenuated tumor-induced suppression of memory CD8^+^ T cell responses.^14^ Deletion of LDL receptor-related protein 5/6 (LRP5/6) in CD11c^+^ DCs abrogated tumor growth with elevated antitumor responses.^15^ Downregulation of the β-catenin or constitutively active glycogen synthase kinase 3 beta (GSK3β) in DCs also enhanced DCs functionality.^16,17^ Besides, Wnt1 fostered transcriptional silencing of CC/CXC chemokines in cDCs, inducing immune resistance in lung adenocarcinoma.^18^ Furthermore, active Wnt/β-catenin signaling pathway dampens CD103^+^ DCs recruitment to the tumors by CCL4 and CCL5.^19,20^

B-cell lymphoma 9 and B-cell lymphoma 9-like (BCL9/BCL9L), functioning as transcriptional coactivators of β-catenin, are crucial in the Wnt/β-catenin signaling pathway.^21^ *BCL9/BCL9L* expression is elevated in various human cancers and correlates with poor prognosis in cancer patients.^22–24^Our previous studies reported that inhibition of BCL9/BCL9L promotes antitumor responses via various mechanisms, involving regulatory T cells (Treg) infiltration, CD8^+^ T cell function, DC infiltration and tumor-associated macrophages.^25–28^ cDC1 have recognized to play an essential role in cross-presentation for CD8^+^ T cell priming and cancer immunotherapy. However, whether targeting BCL9/BCL9L governs the antigen presentation, especially antigen presentation of cDC1 in antitumor immunity is still not completely clear.

Here, we report that inhibition or depletion of BCL9/BCL9L markedly restrained tumor growth and enhanced antitumor responses. Interestingly, targeting BCL9/BCL9L to augment the antigen presentation capacity of cDC1 and boost the infiltration of cDC1 and CD8^+^ T cells into tumors underscores a pivotal role of this approach in bolstering cDC1-mediated antitumor immunity. Therapies aiming at improving antigen presentation might benefit from combination with BCL9 inhibitors.

## Results

### *BCL9* expression is negatively associated with antigen presentation in cancers

**Fig. 1:**
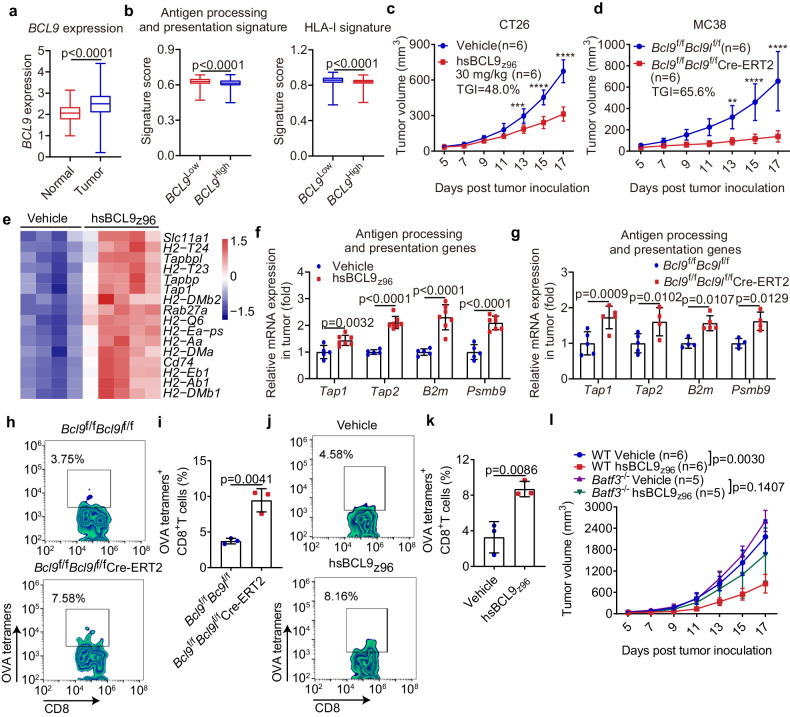
Pharmacological inhibition of BCL9 induces tumor regression and increases antigen presentation. **a** The *BCL9* expression between tumors and normal tissues in TCGA COAD datasets (Normal, *n* = 41; Tumor, *n* = 462). **b** The antigen processing and presentation signature (left) and HLA-I signature (right) between low and high *BCL9* expression (median value) in TCGA COAD datasets (*BCL9*^Low^_,_ *n* = 209; *BCL9*^High^, *n* = 236). **c** Tumor growth of 30 mg/kg hsBCL9_z96_-treated CT26 tumor-bearing mice (*n* = 6). **d** Tumor growth of MC38 tumor-bearing *Bcl9*^f/f^*Bcl9l*^f/f^ mice and *Bcl9*^f/f^*Bcl9l*^f/f^ Cre-ERT2 mice treated i.p. with tamoxifen (1 mg/100 μL) in olive oil on days −7, −6, −5, +1, +6, and +11 post inoculation (*n* = 6). **e** Heatmap of the genes included in the GO:0019882 from 30 mg/kg hsBCL9_z96_-treated CT26 tumors (Vehicle, *n* = 4; hsBCL9_z96_, *n* = 5). **f**, **g** The relative expression of *Tap1*, *Tap2*, *B2m* and *Psmb9* of tumors from hsBCL9_z96_-treated CT26 tumor-bearing mice (**f**) and MC38 tumor-bearing *Bcl9*^f/f^*Bcl9l*^f/f^ Cre-ERT2 mice (**g**) analyzed by qPCR (*n* = 4–7). **h**–**k** Representative plot (**h**, **j**) and quantitative analysis (**i**, **k**) of OVA_257-264_-specific CD8^+^ T cells in TILs of tumors from MC38-OVA tumor-bearing *Bcl9*^f/f^*Bcl9l*^f/f^ Cre-ERT2 mice (**h**, **i**) and hsBCL9_z96_-treated MC38-OVA tumor-bearing mice (**j**, **k**) treated i.p. with tamoxifen (1 mg/100 μL) in olive oil on days −7, −6, −5, +1, +6, and +11 post inoculation and analyzed by flow cytometry (*n* = 3). **l** Tumor growth of C57BL/6 WT (*n* = 6) and *Batf3*^−/−^ mice (*n* = 5) that had been injected subcutaneously with MC38 tumor cells and were treated i.p. with vehicle or 40 mg/kg hsBCL9_z96_ every day for 2 weeks. These data are representative values expressed as the mean ± SD of each group; *n* indicates biological replicate; **p < 0.01; ***p < 0.001; ****p < 0.0001; Unpaired Student’s *t* test (**a**, **b**, **i**, **k**); Two-way ANOVA followed by Bonferroni test (**c**, **d**, **f**, **g**)

Absenting effective antigen presentation is an important reason for immune evasion. Notably, high antigen processing and presentation and HLA-I expression were associated with prolonged overall survival in cancer patients (Supplementary Fig. 1a). Especially, immunotherapy resistance often occurs in microsatellite-stable/microsatellite instability-low (MSS/MSI-L) tumors that have deficient antigen processing and presentation, HLA-I expression and immune infiltration (Supplementary Fig. 1b). We wondered whether oncogenic *BCL9* highly expressed in colon cancer correlates with antigen presentation (Fig. 1a). We analyzed recently published gene expression datasets in tumors.^29^ Interestingly, tumors with high *BCL9* expression lacked antigen processing and presentation, HLA-I expression, and immune infiltration (Fig. 1b and Supplementary Fig. 1c). Similar results were also observed in patients with melanoma and breast cancer (Supplementary Fig. 2a–d). Moreover, cancer patients with high *BCL9* expression had significantly worse overall survival than those with low *BCL9* expression among the tumors exhibiting low immune infiltration, especially among those also displaying Wnt activity (Supplementary Fig. 2e). Collectively, such data indicate that *BCL9* expression is negatively associated with antigen presentation and immune infiltration in cancers. Targeting BCL9 might have beneficial effects in improving resistance to immunotherapy by enhancing antigen presentation.

### Pharmacological inhibition or depletion of BCL9/BCL9L markedly delays tumor growth

As our results showed that elevated *BCL9* expression is associated with diminished antigen presentation in cancers, we explored this supposition in tumor models. To investigate tumor growth driven by oncogenic BCL9, we developed a novel hsBCL9_z96_ that targets the BCL9/β-catenin interaction to inhibit the Wnt/β-catenin signaling pathway. hsBCL9_z96_ was similar to hsBCL9_CT_-24, a BCL9/β-catenin inhibitor reported in our previous study, but had a superior cell penetration ability than hsBCL9_CT_-24.^25^ As a tool of pharmacological inhibition of BCL9, the kinetic constants for the interaction of the protein β-catenin with hsBCL9_z96_ were analyzed by Biacore assay. The kinetic constants were shown in a sensorgram (Equilibrium dissociation constant, *K*_D_ = 3.104 × 10^−8^ M; association rate constant, *K*_a_ = 1.329 × 10^5^ M^−1^s^−1^; dissociation rate constant, *K*_d_ = 0.004126 s^−1^), indicating that hsBCL9_z96_ could excellently interact with β-catenin (Supplementary Fig. 3a). The half-maximal inhibitory concentration (IC_50_) of hsBCL9_z96_ trifluoroacetic acid (TFA) salts for inhibition of the T-cell factor/lymphoid enhancer-binding factor (LEF/TCF) pathway, the major end point mediators of Wnt/Wingless signaling, was only 142.1 nM, indicating that hsBCL9_z96_ could inhibit Wnt/β-catenin signaling in a low dose (Supplementary Fig. 3b). Moreover, the IC_50_ of hsBCL9_z96_ for inhibiting the proliferation of Colo320DM cells was 0.87 μM, which lowed 0.5 μM than that of doxorubicin (Supplementary Fig. 3c). In addition, the cell permeability of hsBCL9_z96_ was approximately 2 times higher than that of hsBCL9_CT_-24 (Supplementary Fig. 3d, e). Collectively, hsBCL9_z96_ exhibits robust inhibition against the interaction between β-catenin and BCL9, as well as shows a favorable profile in vitro.

Furthermore, hsBCL9_z96_ treatment remarkably reduced tumor volumes in subcutaneous implanted CT26 model, which is Wnt/β-catenin signaling-dependent colon carcinoma, with the tumor growth inhibition rate (TGI) of 48.0%, but the body weight also modestly decreased in hsBCL9_z96_-treated tumor-bearing mice (Fig. 1c and Supplementary Fig. 3f). In contrast, histological examination of hearts, livers, lungs, kidneys and spleens from hsBCL9_z96_-treated CT26 tumor-bearing mice indicated that there is no significant toxicity in these organs compared to those in the vehicle control group, except for some intestinal adhesion (Supplementary Fig. 2g). Similarly, hsBCL9_z96_ also demonstrated tumor regression in MC38, B16F10 and 4T1 models, with the TGIs of 56.1%, 45.7% and 33.1%, respectively (Supplementary Fig. 3h–j). In summary, hsBCL9_z96_ demonstrates robust antitumor activity in colon cancer, melanoma and breast cancer models with improved feature of cell permeability, suggesting hsBCL9_z96_ is a good research tool of pharmacological inhibition of BCL9/β-catenin driven Wnt transcriptional activity.

Pharmacological inhibition and genetic depletion are usually used to uncover functional roles of a target protein. To investigate *Bcl9/Bcl9l* genes in mice, we used a *Bcl9*^f/f^*Bcl9l*^f/f^ Cre-ERT2 mouse model, in which *Bcl9/Bcl9l* deletion was induced with tamoxifen in adults. We found that tumor growth was notably decreased in MC38 tumor-bearing *Bcl9*/*Bcl9l* deficiency mice compared with control mice, with a TGI of 65.6% (Fig. 1d). This result indicates that targeting BCL9/BCL9L impairs the development of Wnt/β-catenin signaling-dependent cancers in vivo.

### Targeting BCL9/BCL9L enhances antigen presentation and facilitates antigen-specific CD8^+^ T cell responses

CD4^+^ T cells and CD8^+^ T cells have been implicated in antitumor responses.^30^ hsBCL9_z96_ treatment attenuated the antitumor response in CD8^+^ T cells-depleted group, but not in CD4^+^ T cells-depleted group of CT26 tumor-bearing mice, suggesting that the antitumor responses of hsBCL9_z96_ treatment depends on CD8^+^ T cells (Supplementary Fig. 4a, b). To further investigate the key role of CD8^+^ T cells, we evaluated the frequencies of effector CD8^+^ T cells in hsBCL9_z96_-treated tumors by flow cytometry. Our study revealed that treatment with hsBCL9_z96_ significantly enhanced the proportions of IFN-γ^+^ CD8^+^, Granzyme B^+^ CD8^+^ and Ki67^+^ CD8^+^ T cells in tumor-infiltrating lymphocytes (TILs), as well as in tumor-draining lymph nodes (TdLN) and spleens from hsBCL9_z96_-treated CT26 tumor-bearing mice (Supplementary Fig. 4c–f). Similarly, RNA sequencing (RNA-seq) suggested that TCR signaling pathway was upregulated in hsBCL9_z96_-treated CT26 tumors (Supplementary Fig. 4g). Together, these data indicate that targeting BCL9/BCL9L increases CD8^+^ T cell activation and proliferation.

Antigen presentation is essential for activation and proliferation of CD8^+^ T cells. Our previous bioinformatics analysis indicated that targeting BCL9 might be beneficial for antigen presentation. Consistently, the expression of genes related to antigen processing and presentation was upregulated in hsBCL9_z96_-treated CT26 tumors shown by RNA-seq data (Fig. 1e). Similarly, the expression of genes related to antigen processing and presentation such as *Tap1*, *Tap2*, *B2m*, and *Psmb9*, was also upregulated in tumors from hsBCL9_z96_-treated CT26 tumor-bearing mice and MC38 tumor-bearing *Bcl9*/*Bcl9l* deficiency mice (Fig. 1f, g). Such data indicate that targeting BCL9/BCL9L enhances antigen presentation in tumors.

To evaluate the impact of hsBCL9_z96_ treatment on antigen-specific CD8^+^ T cells in vivo, we established a murine model with MC38 tumor cells overexpressing ovalbumin (OVA) (MC38-OVA). The frequency of OVA_257-264_-specific CD8^+^ T cells was increased in TILs from MC38-OVA tumor-bearing *Bcl9*/*Bcl9l* deficiency mice compared to the control (Fig. 1h, i). Similarly, hsBCL9_z96_ treatment also increased the frequency of OVA_257-264_-specific CD8^+^ T cells in TILs (Fig. 1j, k). Thus, these data indicate that targeting BCL9/BCL9L improves antigen presentation and strengthens antigen-specific CD8^+^ T cell responses.

### Inhibition of BCL9/BCL9L enhances cDC1 activation and facilitates cross-priming of CD8^+^ T cells

**Fig. 2:**
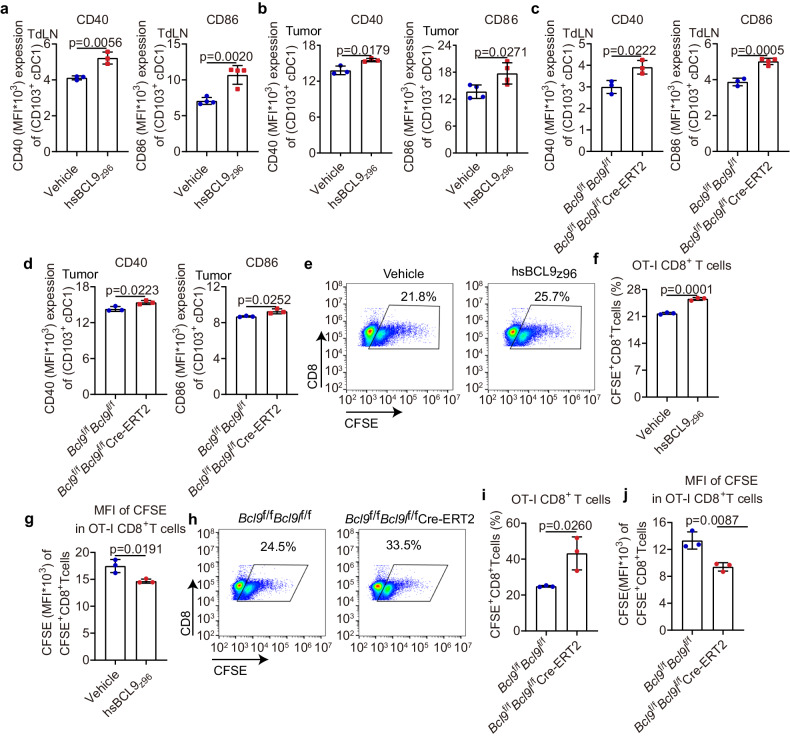
Inhibition of BCL9/BCL9L enhances cDC1 activation and facilitates cross-priming of CD8^**+**^ T cells. **a**, **b** CD40 (left) and CD86 (right) expression by CD103^+^ cDC1 of TdLNs (**a**) and tumors (**b**) from 30 mg/kg hsBCL9_z96_-treated CT26 tumor-bearing mice analyzed by flow cytometry (*n* = 3-4). **c**, **d** CD40 (left) and CD86 (right) expression by CD103^+^ cDC1 of TdLNs (**c**) and tumors (**d**) from MC38 tumor-bearing *Bcl9*^f/f^*Bcl9l*^f/f^ Cre-ERT2 mice treated i.p. with tamoxifen (1 mg/100 μL) in olive oil on days −7, −6, −5, +1, +6, and +11 post inoculation analyzed by flow cytometry (*n* = 3–4). **e** The representative plot of OT-I CD8^+^ T cells in TdLNs from hsBCL9_z96_-treated MC38-OVA tumor-bearing mice analyzed by flow cytometry. **f** and **g** Quantitative analysis of the percentage of OT-I CD8^+^ T cells (**f**) and CFSE dilution of OT-I CD8^+^ T cells (mean fluorescent intensity, MFI) (**g**) based on the result of (**e**) (*n* = 3). **h** The representative plot of OT-I CD8^+^ T cells in TdLNs from MC38-OVA tumor-bearing *Bcl9*^f/f^*Bcl9l*^f/f^ Cre-ERT2 mice treated i.p. with tamoxifen (1 mg/100 μL) in olive oil on days −7, −6, −5, +1, and +6 post inoculation analyzed by flow cytometry. **i**, **j** Quantitative analysis of the percentage of OT-I CD8^+^ T cells (**i**) and CFSE dilution of OT-I CD8^+^ T cells (**j**) based on the result of (**h**) (*n* = 3). These data are representative values expressed as the mean ± standard deviation (SD) for each group, derived from three independent experiments; “*n*” denotes the number of biological replicates. An unpaired Student’s *t* test was used for statistical analysis of the data in groups **a**–**d**, **f**, **g**, **i**, and **j**

cDC1 is a key player in presenting tumor antigens to elicit CD8^+^ T cell-mediated antitumor immunity. The maturation and activation of DC is the primary step for antigen presentation. Our results indicated that targeting BCL9/BCL9L enhances antigen presentation. We wondered if this is mediated by upregulating of cDC1 activation. To investigate this hypothesis, we used *Batf3*^−/−^ mice and found that increased anti-tumor immunity upon *Bcl9*/*Bcl9l* blockade was lost in cDC1-deficiency mice (Fig. 1l). We blocked XCL1/XCR1 signaling using anti-XCL1. Our results showed that cDC1 accumulation was not changed in hsBCL9_z96_-treated mice after blocking the XCL1-XCR1 axis (Supplementary Fig. 5a). We assessed the expression of activation markers CD40 and CD86 in TdLNs and tumors from hsBCL9_z96_-treated CT26 tumor-bearing mice by flow cytometry. Increased expression of these activation markers on cDC1 was observed in this model, as well as in MC38 tumor-bearing *Bcl9*/*Bcl9l* deficiency mice (Fig. 2a–d). In summary, *Bcl9*/*Bcl9l* deficiency in cDC1 promoted CD8^+^ T cell-mediated immunity which is dependent on XCL1-XCR1 axis.

In addition, staining results of SIINFEKL-H2Kb complexes on cDC1 of TDLNs isolated from OVA-expressing tumor-bearing mice showed an increase in cross-presentation among hsBCL9_z96_-treated MC38-OVA tumor-bearing mice or *Bcl9*^f/f^*Bcl9l*^f/f^ Cre-ERT2 MC38-OVA tumor-bearing mice compared with vehicle or *Bcl9*^*f/f*^*Bcl9l*^*f/f*^ *mice*, respectively (Supplementary Fig. 5b, c).

To assess the impact of this activation directly, we then sorted naïve CD8^+^ T cells from OT-I mice, labeled them with CFSE and injected intravenously into mice that had been implanted with MC38-OVA tumors. Enhanced proliferation of OT-I CD8^+^ T cells was observed in TdLNs from hsBCL9_z96_-treated MC38-OVA tumor-bearing mice (Fig. 2e–g). Similar results were also obtained in TdLNs from MC38-OVA tumor-bearing *Bcl9*/*Bcl9l* deficiency mice (Fig. 2h–j).

To determine the cross-presentation activity upon BCL9 inhibition, we used in vitro and in vivo approaches. CD8^+^ T cell in vitro proliferation was increased in cross-presentation of CD11c^+^ DC-CD8^+^ T cells in both hsBCL9_Z96_ tumor model and *Bcl9/Bcl9l* KO tumor model compared with vehicle and wild-type (WT) respectively (Supplementary Fig. 5d, e). In ELISPOT assay, in vitro co-culture of CD11c^+^ DCs from TDLNs and naïve CD8^+^ T derived from OT-I mice, IFN-γ secretion was increased in CD11c^+^ DCs from hsBCL9z96-treated MC38-OVA-expressing tumors compared with vehicle treatment; IFN-γ secretion was also increased in CD11c^+^ DCs from MC38-OVA tumor-bearing *Bcl9*^f/f^*Bcl9l*^f/f^ Cre-ERT2 mice compared with *Bcl9*^f/f^*Bcl9l*^f/f^ mice (Supplementary Fig. 5f, g). We then purified CD8^+^ T cells from TDLNs and stimulated them with of 10 μg/ml of OVA-I peptide for 24 h to determine IFN-γ-producing cells by ELISPOT assay. Upon OVA-I peptide stimulation, IFN-γ-producing cells were increased in CD8^+^ T cells from hsBCL9z96-treated MC38-OVA tumor-bearing mice. Similar results were obtained in *Bcl9*^f/f^*Bcl9l*^f/f^ Cre-ERT2 MC38-OVA tumor-bearing mice (Supplementary Fig. 5h, i). In summary, BCL9 inhibition promotes CD8^+^ T cell proliferation and IFN-γ secretion as well as cross-presentation in MC38-OVA tumor-bearing models.

Collectively, the data indicates that inhibition of BCL9/BCL9L promotes cDC1 activation and facilitates cross-priming of CD8^+^ T cells.

### Single-cell transcriptional profiling of CD8^+^ T cells and cDC1 of tumors and TdLNs from B16-OVA tumor-bearing *Bcl9/Bcl9l* deficiency mice

**Fig. 3:**
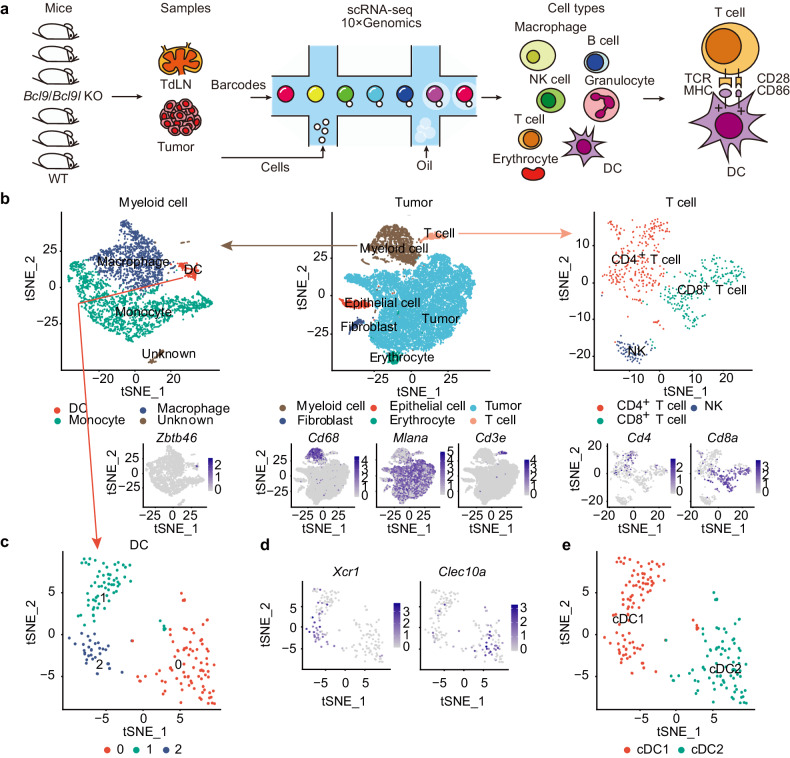
Single-cell transcriptional profiling of CD8^**+**^ T cells and cDC1 in tumors and TdLNs from B16-OVA tumor-bearing *Bcl9/Bcl9l* deficiency mice. **a** Illustration of experiment and analysis process of single-cell transcriptional analysis. **b** TSNE plots of clustering process and marker genes (*Zbtb46* for DCs, *Cd68* for myeloid cells, *Mlana* for B16-OVA tumor cells, *Cd3e* for T cells, *Cd4* for CD4^+^ T cells and *Cd8a* for CD8^+^ T cells) in tumors from B16-OVA tumor-bearing *Bcl9*^f/f^*Bcl9l*^f/f^ mice and *Bcl9*^f/f^*Bcl9l*^f/f^ Cre-ERT2 mice treated i.p. with tamoxifen (1 mg/100 μL) in olive oil on days −7, −6, −5, +1, +6 and +11 post inoculation. **c**–**e** TSNE plots of DC reclustering (**c**, **e**) and marker genes (*Xcr1* for cDC1 and *Clec10a* for cDC2) (**d**) in tumors from B16-OVA tumor-bearing *Bcl9*^f/f^*Bcl9l*^f/f^ mice and *Bcl9*^f/f^*Bcl9l*^f/f^ Cre-ERT2 mice treated *i.p*. with tamoxifen (1 mg/100 μL) in olive oil on days −7, −6, −5, +1, +6, and +11 post inoculation

To precisely investigate how BCL9/BCL9L governs the functions of cDC1, we performed single-cell sequencing (scRNA-seq) of tumors and TdLNs from B16-OVA tumor-bearing *Bcl9*/*Bcl9l* deficiency mice (Fig. 3a). All cells were clustered according to t-SNE (t-distributed Stochastic Neighbor Embedding), and then myeloid cells (marked by *Cd68*) and T cells (marked by *Cd3e*) were extracted for further clustering (Fig. 3b and Supplementary Fig. 6a). T cells were mainly divided into 2 clusters, including CD8^+^ T cells (marked by *Cd8a*) and CD4^+^ T cells (marked by *Cd4*) (Fig. 3b, and Supplementary Fig. 6a). Additionally, DCs (marked by *Zbtb46*) were separated from the myeloid cells and then clustered into cDC1 (marked by *Xcr1*) and cDC2 (marked by *Clec10a*) (Fig. 3c-e and Supplementary Fig. 6b–d). The expression profiles of DC-related genes and differentially expressed genes in the tumors and TdLNs of DC subclusters were shown (Supplementary Fig. 7a–d). In summary, we successfully identified the CD8^+^ T cells and cDC1 populations in tumors and TdLNs from B16-OVA tumor-bearing *Bcl9/Bcl9l* deficiency mice using scRNA-seq.

### *Bcl9/Bcl9l* deficient cDC1 are superior to WT cDC1 in activation, antigen presentation and cross-priming of CD8^+^ T cells

**Fig. 4:**
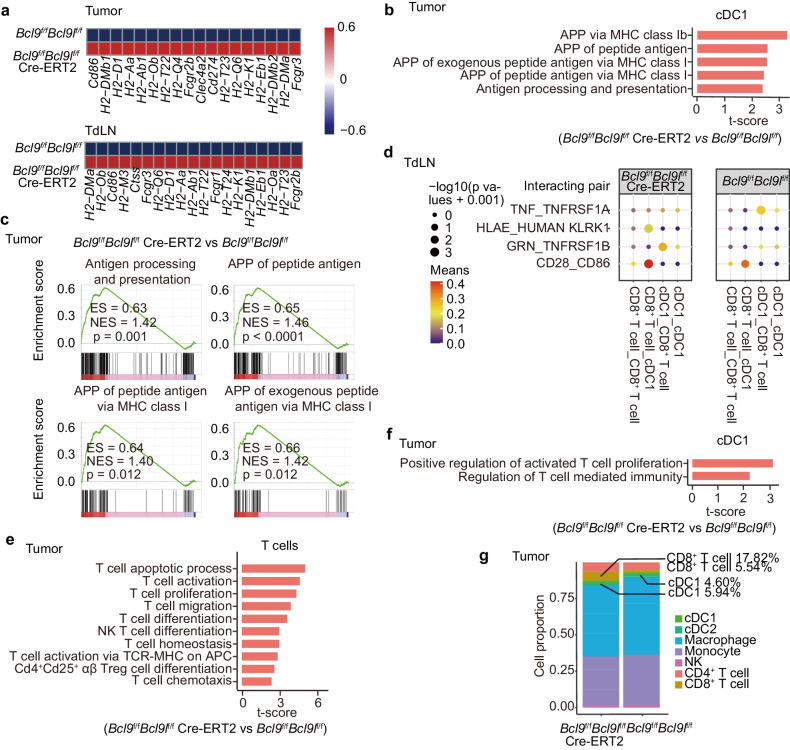
*Bcl9/Bcl9l* deficient cDC1 are superior to WT cDC1 in activation, antigen presentation and cross-priming of CD8^**+**^ T cells. **a** Expression of genes related to cDC1 maturation and antigen presentation in tumors and TdLNs from B16-OVA tumor-bearing *Bcl9*^f/f^*Bcl9l*^f/f^ mice and *Bcl9*^f/f^*Bcl9l*^f/f^ Cre-ERT2 mice treated i.p. with tamoxifen (1 mg/100 μL) in olive oil on days −7, −6, −5, +1, +6 and +11 post inoculation. **b**, **c** GSVA (**b**) and GSEA (**c**) of cDC1 for gene sets in tumors from B16-OVA tumor-bearing *Bcl9*^f/f^*Bcl9l*^f/f^ mice and *Bcl9*^f/f^*Bcl9l*^f/f^ Cre-ERT2 mice treated i.p. with tamoxifen (1 mg/100 μL) in olive oil on days −7, −6, −5, +1, +6, and +11 post inoculation. APP, antigen processing and presentation. **d** Cell communication analysis of cDC1 and CD8^+^ T cells in TdLNs from B16-OVA tumor-bearing *Bcl9*^f/f^*Bcl9l*^f/f^ mice and *Bcl9*^f/f^*Bcl9l*^f/f^ Cre-ERT2 mice treated i.p. with tamoxifen (1 mg/100 μL) in olive oil on days −7, −6, −5, +1, +6, and +11 post inoculation. **e** GSVA of T cells in tumors from B16-OVA tumor-bearing *Bcl9*^f/f^*Bcl9l*^f/f^ mice and *Bcl9*^f/f^*Bcl9l*^f/f^ Cre-ERT2 mice treated i.p. with tamoxifen (1 mg/100 μL) in olive oil on days −7, −6, −5, +1, +6, and +11 post inoculation. The gene sets related to T cell activation and function are shown. T cell activation via TCR-MHC on APC, is the abbreviation for T cell activation via TCR contact with Ag (antigen) bound to MHC on APC. **f** GSVA of cDC1 in tumors from B16-OVA tumor-bearing *Bcl9*^f/f^*Bcl9l*^f/f^ mice and *Bcl9*^f/f^*Bcl9l*^f/f^ Cre-ERT2 mice treated i.p. with tamoxifen (1 mg/100 μL) in olive oil on days −7, −6, −5, +1, +6, and +11 post inoculation. The gene sets related to regulation of T cells are shown. **g** Cell fraction of TILs from tumors of B16-OVA tumor-bearing *Bcl9*^f/f^*Bcl9l*^f/f^ mice and *Bcl9*^f/f^*Bcl9l*^f/f^ Cre-ERT2 mice treated i.p. with tamoxifen (1 mg/100 μL) in olive oil on days −7, −6, −5, +1, +6, and +11 post inoculation

To further investigate how BCL9/BCL9L regulates the functions of cDC1, we explored the transcriptional differences of CD8^+^ T cells and cDC1 in TdLNs and tumors from B16-OVA tumor-bearing *Bcl9*/*Bcl9l* deficiency mice using single-cell transcriptomics analysis. We analyzed transcriptional changes and signaling enrichment of cDC1 in tumors and TdLNs from B16-OVA tumor-bearing *Bcl9*/*Bcl9l* deficiency mice. Enhancement of activation and antigen presentation was observed in *Bcl9/Bcl9l* deficiency cDC1 of tumors and TdLNs, consistently with the results that inhibition of BCL9/BCL9L induced cDC1 activation (Fig. 4a–c). Interacting pairs of HLAE_HUMAN-KLRK1 showed stronger signals in TdLNs from *Bcl9*/*Bcl9l* deficiency mice than those from the control mice, suggesting enhanced antigen presentation and interaction between cDC1 and CD8^+^ T cells (Fig. 4d). Importantly, T cell activation was enhanced in tumors and TdLNs from B16-OVA tumor-bearing *Bcl9*/*Bcl9l* deficiency mice (Fig. 4e and Supplementary Fig. 8a). Consistently, positive regulation of T cell proliferation in cDC1 was also upregulated in tumors of B16-OVA tumor-bearing *Bcl9*/*Bcl9l* deficiency mice, supporting that cDC1 primed CD8^+^ T cells (Fig. 4f and Supplementary Fig. 8b). In line with this result, CD8^+^ T cells infiltration was markedly increased in B16-OVA tumor-bearing *Bcl9*/*Bcl9l* deficiency mice compared with control mice (Fig. 4g). Together, these data indicate that *Bcl9/Bcl9l* -deficient cDC1 outperform WT cDC1 in activation, antigen presentation and cross-priming of CD8^+^ T cells.

### *Bcl9/Bcl9l* deficiency facilitates cDC1 activation and antigen presentation via TAK1/NF-κB/IRF1 axis

**Fig. 5:**
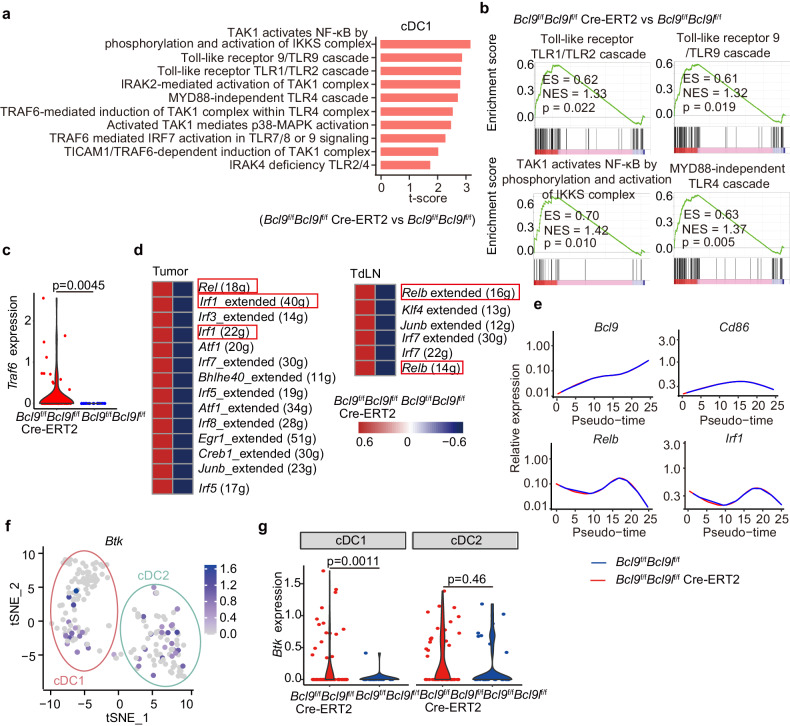
*Bcl9/Bcl9l* deficiency facilitates cDC1 activation and antigen presentation via TAK1/NF-κB/IRF1 axis. **a**–**c** GSVA (**a**), GSEA (**b**) and *Traf6* expression (**c**) of cDC1 in tumors from B16-OVA tumor-bearing *Bcl9*^f/f^*Bcl9l*^f/f^ mice and *Bcl9*^f/f^*Bcl9l*^f/f^ Cre-ERT2 mice treated i.p. with tamoxifen (1 mg/100 μL) in olive oil on days −7, −6, −5, +1, +6, and +11 post inoculation. **d** Transcription factors that were activated in cDC1 of tumors and TdLNs from B16-OVA tumor-bearing *Bcl9*^f/f^*Bcl9l*^f/f^ mice and *Bcl9*^f/f^*Bcl9l*^f/f^ Cre-ERT2 mice treated i.p. with tamoxifen (1 mg/100 μL) in olive oil on days −7, −6, −5, +1, +6, and +11 post inoculation. **e** Pseudo-time analysis of cDC1 in tumors from B16-OVA tumor-bearing *Bcl9*^f/f^*Bcl9l*^f/f^ mice and *Bcl9*^f/f^*Bcl9l*^f/f^ Cre-ERT2 mice treated *i.p*. with tamoxifen (1 mg/100 μL) in olive oil on days −7, −6, −5, +1, +6, and +11 post inoculation. The trends of *Bcl9*, *Cd86*, *Relb*, and *Irf1* expression through time are shown. **f** TSNE plots of *Btk* in DC. **g** The expression of *Btk* in cDC1 and cDC2 between tumors from B16-OVA tumor-bearing *Bcl9*^f/f^*Bcl9l*^f/f^ and *Bcl9*^f/f^*Bcl9l*^f/f^ Cre-ERT2 mice treated i.p. with tamoxifen (1 mg/100 μL) in olive oil on days −7, −6, −5, +1, +6, and +11 post inoculation. These data are representative charts or values expressed as the mean ± SD of each group; Unpaired Student’s *t* test (**c**, **g**)

To study the underlying mechanism of activation and antigen presentation of cDC1 by targeting BCL9/BCL9L, we performed gene set variation analysis (GSVA) and gene set enrichment analysis (GSEA). We found that Toll-like receptor (TLR) signaling and especially transforming growth factor-β-activated kinase1 (TAK1) mediated NF-κB signaling were enriched in *Bcl9/Bcl9l* deficient cDC1 (Fig. 5a, b). Moreover, TNF receptor associated factor 6 (TRAF6), which is involved in these pathways was also evidently upregulated in *Bcl9/Bcl9l* deficient cDC1 (Fig. 5c). Stimulation of cells with TLR ligands activates intracellular TAK1 mediated NF-κB signaling that contributes to DC activation.^31–33^ To confirm the TAK1 functions in DCs, we calculated the GSVA scores between *Map3k7* (*Tak1*) knocking-out (KO) and WT DCs using data from the dataset GSE34417.^34^ Antigen presentation and NF-κB signaling were downregulated in *Map3k7* (*Tak1*) KO DCs, which confirms the importance of TAK1 in NF-κB pathway and antigen presentation of DCs (Supplementary Fig. 9a). Such data indicated that *Bcl9*/*Bcl9l* deficient cDC1 might facilitate activation through TLR/TAK1/NF-κB signaling. To explore this hypothesis, we performed Pearson correlation analysis between *BCL9*, *CD86*, TLR4/TAK1 GSVA scores and TAK1/NF-κB GSVA scores for patients with SKCM, BRCA, and COAD in TCGA datasets bearing high cDC1 scores. Consistently, *BCL9* was negatively correlated with *CD86*, TLR4/TAK1 signaling and TAK1/NF-κB signaling, while *CD86* was positively correlated with TLR4/TAK1 signaling and TAK1/NF-κB signaling in these datasets (Supplementary Fig. 9b–f). Together, the data demonstrate that *Bcl9/Bcl9l* deficient cDC1 promotes activation and antigen presentation through TLR/TAK1/NF-κB signaling. In addition, interferon regulatory factor 1 (IRF1) is a transcriptional regulator, which is involved in NF-κB signaling, has been linked to cDC1 activation recently.^35^ We wondered if *Bcl9/Bcl9l* deficient cDC1 boost activation and antigen presentation through TLR/TAK1/NF-κB/IRF1 axis. To investigate this, we assessed the expression of *Rel*, *Relb* and *Irf1* of cDC1 in TdLNs and tumors from B16-OVA tumor-bearing *Bcl9*/*Bcl9l* deficiency mice using SCENIC analysis. In line with our conjecture, *Rel*, *Relb* and *Irf1* were upregulated in *Bcl9/Bcl9l* deficient cDC1 (Fig. 5d). Moreover, the expression trend of *Bcl9* opposed those of *Cd86*, *Relb*, and *Irf1* in cDC1 shown by pseudo-time analysis (Fig. 5e and Supplementary Fig. 10a). In addition, we found that the expression of *Irf1* and antigen processing and presentation genes (*Psma5*, *Psmb9, Psmb3*, *Ube2f* and *Ncf4*) were also significantly elevated in *Bcl9/Bcl9l* deficient cDC1 (Supplementary Fig. 10b–g). Moreover, we also studied the expression of phospho-TAK1, phospho-NF-κB p65 and IRF1 of intratumoral cDC1 by flow cytometry. The expression of phospho-TAK1, phospho-NF-κB p65, and IRF1 of intratumoral cDC1 were upregulated from hsBCL9_z96_-treated CT26 tumor-bearing mice, as well as from MC38 tumor-bearing *Bcl9*/*Bcl9l* deficiency mice (Supplementary Fig. 10h–m). Furthermore, we demonstrated that TAK1/NF-κB/IRF1 signaling was also activated in cDC1 from TdLNs of MC38 tumor-bearing *Bcl9*/*Bcl9l* deficiency mice using multiplex immunofluorescence (Supplementary Fig. 11a). In summary, these data indicated that *Bcl9/Bcl9l* deficient cDC1 outperform WT cDC1in activation and antigen presentation via TAK1/NF-κB/IRF1 signaling.

### *Bcl9/Bcl9l* deficient cDC1 activate TAK1/NF-κB/IRF1 signaling by upregulating BTK expression

Next, we investigated how *Bcl9/Bcl9l* deficient cDC1 activate TAK1/NF-κB/IRF1 signaling. Bruton’s tyrosine kinase (BTK), a part of the Tec family of kinases, plays a crucial role in the development and activation of B cells via B-cell receptor (BCR) and TLR signaling, especially can interact with TLR2, TLR4, TLR6 and TLR9 that activate downstream NF-κB signaling.^36–38^ BTK expression is not limited to B cells, other myeloid cell lineages, including DCs, macrophages, monocytes and neutrophils, can also express BTK, although the function of BTK in these myeloid cells is little known.^38–40^ BTK is reported to affect DCs differentiation and function.^41,42^ We found that *Btk* was expressed in both cDC1 and cDC2 by using scRNA-seq (Fig. 5f). We wondered if *Bcl9/Bcl9l* deficient cDC1 activates TAK1/NF-κB/IRF1 signaling by regulating BTK expression. Surprisingly, we found that *Btk* expression was remarkably higher in *Bcl9*/*Bcl9l* deficient cDC1 than WT cDC1, while there was not a significant difference between *Bcl9*/*Bcl9l* deficient cDC2 and WT cDC2, indicating BTK plays a key role in *Bcl9*/*Bcl9l* deficient cDC1 (Fig. 5g). To further investigate this, we performed Pearson correlation analysis between *BCL9*, *BTK*, TLR4/TAK1 GSVA scores and TAK1/NF-κB GSVA scores in patients with SKCM, BRCA and COAD from TCGA datasets bearing high cDC1 scores. Consistently, *BTK* was negatively correlated with *BCL9*, while *BTK* was positively correlated with *CD86*, TLR4/TAK1 signaling pathway and TAK1/NF-κB signaling in these datasets (Supplementary Fig. 12a–d). In conclusion, these data indicate that *Bcl9/Bcl9l*-deficient cDC1 activates TAK1/NF-κB/IRF1 signaling by upregulating BTK expression.

### Targeting BCL9/BCL9L increases cDC1 accumulation in tumors through XCL1-XCR1 axis

**Fig. 6:**
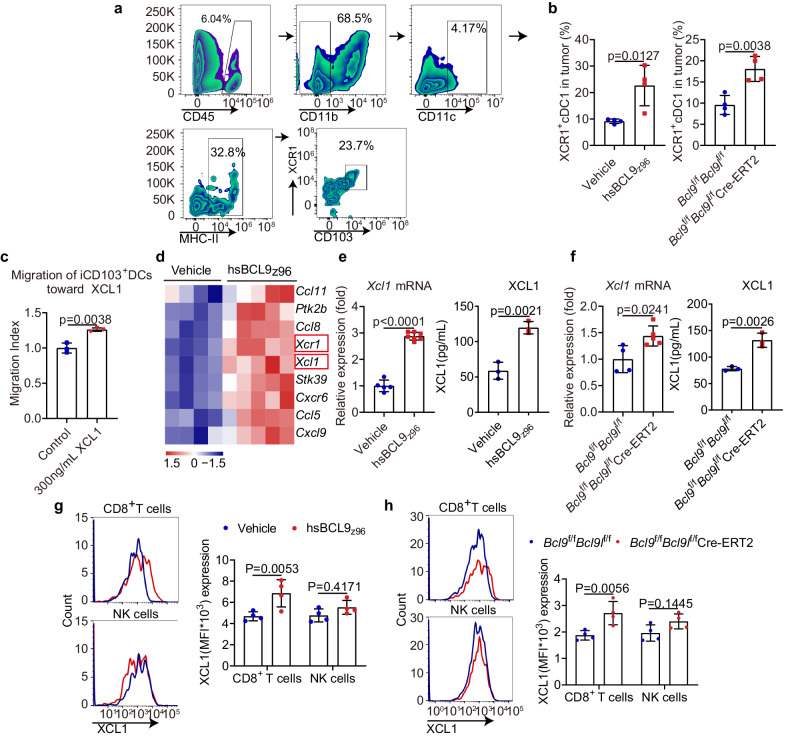
Targeting of BCL9/BCL9L increases cDC1 accumulation in tumors through XCL1-XCR1 axis. **a** Gating strategy of XCR^+^ cDC1 (CD45^+^ CD11b^−^ CD11c^+^ MHC-II^+^ CD103^+^ XCR1^+^) in TILs. **b** The XCR^+^ cDC1 in TILs of 30 mg/kg hsBCL9_z96_-treated CT26 tumor-bearing mice (left) and MC38 tumor-bearing *Bcl9*^f/f^*Bcl9l*^f/f^Cre-ERT2 mice (right) treated i.p. with tamoxifen (1 mg/100 μL) in olive oil on days −7, −6, −5, +1, +6, and +11 post inoculation were analyzed by flow cytometry (*n* = 4). **c** iCD103^+^ DC migration toward XCL1 for 3 h by trans well assay (*n* = 3). **d** Heatmap of the genes included in GO:0070098 of 30 mg/kg hsBCL9_z96_-treated CT26 tumors (vehicle, *n* = 4; hsBCL9_z96_, *n* = 5). **e**, **f** *Xcl1* mRNA (left) and XCL1 protein (right) levels in tumors from 30 mg/kg hsBCL9_z96_-treated CT26 tumor-bearing mice (**e**) and MC38 tumor-bearing *Bcl9*^f/f^*Bcl9l*^f/f^Cre-ERT2 mice (**f**) treated i.p. with tamoxifen (1 mg/100 μL) in olive oil on days −7, −6, −5, +1, +6, and +11 post inoculation were analyzed by qPCR and ELISA, respectively (*n* = 4-5). **g**, **h** Representative plot (left) and quantitative analysis (right) of XCL1 expression of CD8^+^ T cells and NK cells in TILs from 30 mg/kg hsBCL9_z96_-treated CT26 tumor-bearing mice analyzed by flow cytometry (*n* = 4). **g** Representative plot (left) and quantitative analysis (right) of XCL1 expression among CD8^+^ T cells and NK cells in TILs from MC38 tumor-bearing *Bcl9*^f/f^*Bcl9l*^f/f^ Cre-ERT2 mice treated i.p. with tamoxifen (1 mg/100 μL) in olive oil on days −7, −6, −5, +1, +6, and +11 post inoculation were analyzed by flow cytometry (*n* = 4). **h** Results are presented as the mean ± standard deviation (SD) for each group, derived from three independent experiments; “*n*” denotes the number of biological replicates; Unpaired Student’s *t* test (**b**, **c**, **e**, **f**); Two-way ANOVA followed by Bonferroni test (**g**, **h**)

cDC1 activation and cDC1 numbers are crucial to antitumor immunity. In human cancers, there are increasing evidence that intratumoral cDC1 are associated with the improved prognosis and responses to cancer immunotherapy.^6,9^ Having established that targeting BCL9/BCL9L facilitates activation and antigen presentation of cDC1, we guessed that targeting BCL9/BCL9L may promote cDC1 accumulation in tumors owing to the increased numbers of intratumoral cDC1 from B16-OVA tumor-bearing *Bcl9*/*Bcl9l* deficiency mice shown by single-cell analysis (Fig. 4g). XCR1, which is expressed specifically on cDC1, could binds to XCL1, regulating cDC1 recruitment.^9^ To investigate our hypothesis, we established a gating strategy to identify XCR1^+^ cDC1 by flow cytometry (Fig. 6a). We found that cDC1 numbers are increased in tumors from hsBCL9_z96_-treated CT26 tumor-bearing mice, as well as MC38 tumor-bearing *Bcl9*/*Bcl9l* deficiency mice (Fig. 6b). To study the mechanism by which targeting BCL9/BCL9L mediates cDC1 migration, we performed differential gene expression analysis. We found that *Xcl1* and *Xcr1* that control cDC1 migration were notably upregulated in tumors from hsBCL9_z96_-treated CT26 tumor-bearing mice (Fig. 6c, d). Identically, *Xcl1* mRNA and XCL1 protein levels were increased markedly in these tumors using qPCR, as well as tumors from MC38 tumor-bearing *Bcl9*/*Bcl9l* deficiency mice (Fig. 6e, f). Such data indicate that targeting BCL9/BCL9L increases cDC1 accumulation in tumors through XCL1-XCR1 axis.

XCL1 is mainly produced by activated CD8^+^ T cells and NK cells.^9^ To identify the source of XCL1, we analyzed the XCL1 expression in these cells. We found that XCL1 expression by intratumoral CD8^+^ T cells was upregulated in hsBCL9_z96_-treated CT26 tumor-bearing mice and MC38 tumor-bearing *Bcl9*/*Bcl9l* deficiency mice (Fig. 6g, h). These data indicate that targeting BCL9/BCL9L enhances XCL1 production by activating CD8^+^ T cells and subsequently promotes the recruitment of XCR1^+^ cDC1 into tumor sites.

### Targeting BCL9/BCL9L results in CD8^+^ T cell accumulation in tumors through CXCL9-CXCR3 axis

**Fig. 7:**
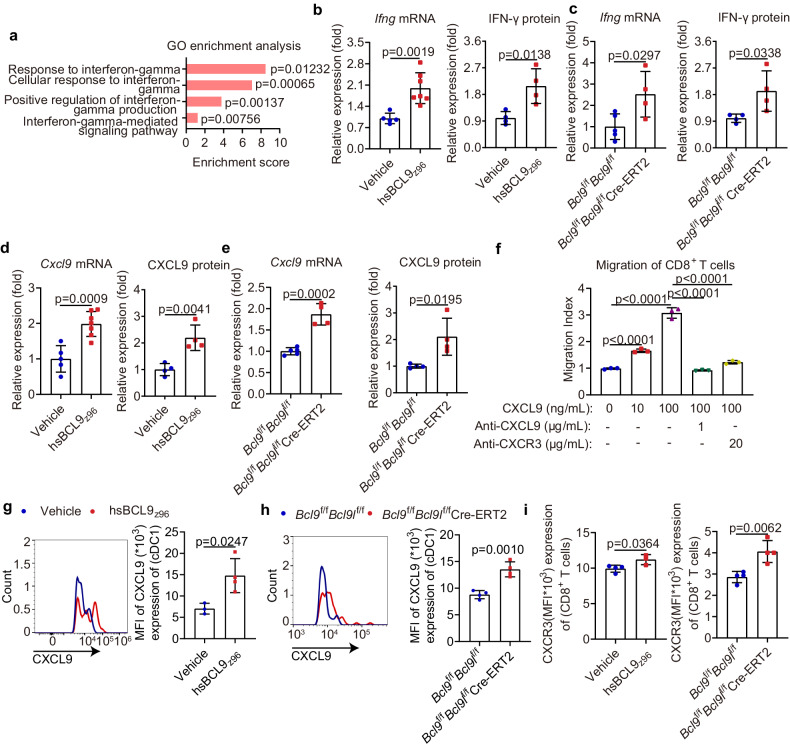
Targeting BCL9/BCL9L results in CD8^**+**^ T cells accumulation in tumors through CXCL9-CXCR3 axis. **a** Significantly upregulated GO terms related to IFN-γ response of 30 mg/kg hsBCL9_z96_-treated CT26 tumors are depicted (vehicle, *n* = 4; hsBCL9_z96_, *n* = 5). **b** and **c** Relative *Ifng* mRNA (left) and IFN-γ protein (right) levels in tumors from 30 mg/kg hsBCL9_z96_-treated CT26 tumor-bearing mice (**b**) and MC38 tumor-bearing *Bcl9*^f/f^*Bcl9l*^f/f^ Cre-ERT2 mice (**c**) treated i.p. with tamoxifen (1 mg/100 μL) in olive oil on days −7, −6, −5, +1, +6, and +11 post inoculation analyzed by qPCR and ELISA, respectively (*n* = 4–7). **d**, **e** Relative *Cxcl9* mRNA (left) and CXCL9 protein (right) expression of tumors from 30 mg/kg hsBCL9_z96_-treated CT26 tumor-bearing mice (**d**) and MC38 tumor-bearing *Bcl9*^f/f^*Bcl9l*^f/f^ Cre-ERT2 mice (**e**) treated i.p. with tamoxifen (1 mg/100 μL) in olive oil on days −7, −6, −5, +1, +6, and +11 post inoculation analyzed by qPCR and ELISA, respectively (*n* = 4-7). **f** Assessment of CD8^+^ T cell migration toward CXCL9 or with the indicated doses of antibodies or chemokine for 4 h by trans well assay (*n* = 3). **g** Representative plot (left) and quantitative analysis (right) of CXCL9 expression in cDC1 of tumors from 30 mg/kg hsBCL9_z96_-treated CT26 tumor-bearing mice analyzed by flow cytometry (*n* = 3–4). **h** Representative plot (left) and quantitative analysis (right) of CXCL9 expression in cDC1 of tumors from MC38 tumor-bearing *Bcl9*/*Bcl9l* deficiency mice analyzed by flow cytometry (*n* = 4). **i** The expression of CXCR3 in CD8^+^ T cells of tumors from 30 mg/kg hsBCL9_z96_-treated CT26 tumor-bearing mice (left) and MC38 tumor-bearing *Bcl9*^f/f^*Bcl9l*^f/f^ Cre-ERT2 mice (right) treated i.p. with tamoxifen (1 mg/100 μL) in olive oil on days −7, −6, −5, +1, +6, and +11 post inoculation analyzed by flow cytometry (*n* = 4). Results are presented as the mean ± standard deviation (SD) for each group, derived from three independent experiments; “*n*” denotes the number of biological replicates; Unpaired Student’s *t* test (**b**–**e**, **g**–**i**); One-way ANOVA followed by Bonferroni test (**f**)

Having established that targeting BCL9/BCL9L facilitates cDC1 activation and tumor infiltration, we next examined how cDC1 contribute to CD8^+^ T cell responses in tumors after the inhibition of BCL9. Using RNA-seq data, we found that IFN-γ response was upregulated in hsBCL9_z96_-treated CT26 tumors compared with vehicle control (Fig. 7a). Similarly, increased *Ifng* mRNA and IFN-γ protein levels were observed in tumors from hsBCL9_z96_-treated CT26 tumor-bearing mice and MC38 tumor-bearing *Bcl9*/*Bcl9l* deficiency mice (Fig. 7b, c). The mRNA and protein levels of the IFN-γ-induced chemokine CXCL9 were also upregulated in tumors from hsBCL9_z96_-treated CT26 tumor-bearing mice and MC38 tumor-bearing *Bcl9*/*Bcl9l* deficiency mice (Fig. 7d, e). cDC1 could produce CXCL9 to recruit effector CXCR3^+^ CD8^+^ T cells into the TME through CXCL9-CXCR3 axis (Fig. 7f).^43^ We wondered if CXCL9 produced from intratumoral cDC1 promotes CD8^+^ T cells infiltration. We thus evaluated CXCL9 expression of cDC1 and CXCR3 expression of CD8^+^ T cells in tumors by flow cytometry. Consistently, the CXCL9 expression by cDC1 and CXCR3 expression by CD8^+^ T cells were upregulated in tumors from hsBCL9_z96_-treated CT26 tumor-bearing mice, as well as from MC38 tumor-bearing *Bcl9*/*Bcl9l* deficiency mice (Fig. 7g–i). In support of these findings, we characterized the datasets of cancer patients from TCGA with either low or high *BCL9* mRNA expression. We found that both *Ifng* signature and CD8^+^ T cells infiltration were increased in low *BCL9* expression patients with COAD, SKCM and BRCA from TCGA datasets compared to those from high *BCL9* expression group (Supplementary Fig. 13a, b). In summary, targeting BCL9/BCL9L promotes the infiltration of CD8^+^ T cells into tumors via the CXCL9-CXCR3 axis.

### Targeting BCL9/BCL9L sensitizes tumors to immune checkpoint blockade therapy

**Fig. 8:**
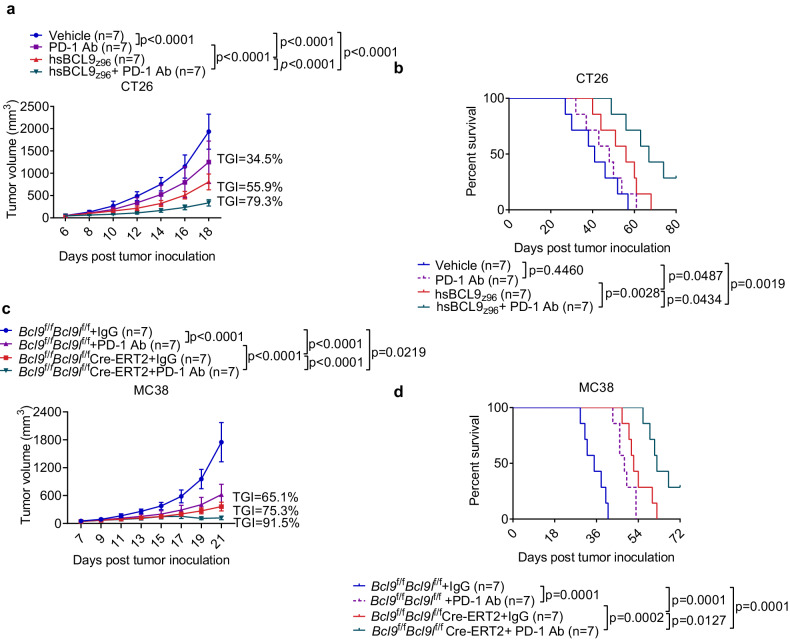
Targeting BCL9/BCL9L sensitizes tumors to immune checkpoint blockade therapy. **a**, **b** Tumor growth (a) and survival (b) of CT26 tumor-bearing mice treated i.p. with vehicle, 30 mg/kg hsBCL9_z96_, 10 mg/kg anti-PD-1 or combinational therapy (*n* = 5-7). **c**, **d** Tumor growth (**c**) and survival (**d**) of MC38 tumor-bearing *Bcl9*^f/f^*Bcl9l*^f/f^ mice and *Bcl9*^f/f^*Bcl9l*^f/f^ Cre-ERT2 mice. Individual mice were treated with tamoxifen on days −7, −6, −5, +1, +6, and +11 post inoculation. For anti-PD-1 combination therapy, individual mice were treated with 10 mg/kg anti-PD-1 every 3 days (*n* = 5). Results are presented as the mean ± standard deviation (SD) for each group, derived from three independent experiments; *n* indicates biological replicate; Two-way ANOVA followed by Bonferroni test (**a**, **c**); One-way ANOVA followed by Bonferroni test (**b**, **d**)

Our studies showed that targeting BCL9/BCL9L facilitates antigen presentation and tumor infiltration of cDC1, as well as boosts tumor infiltration of CD8^+^ T cells, indicating a critical role of targeting BCL9/BCL9L in overcoming immunotherapy resistance. To evaluate this point, we combined hsBCL9_z96_ treatment with anti-PD-1 treatment in established CT26 tumor-bearing mice. The CT26 tumors showed a stronger response to combination therapy than to hsBCL9_z96_ monotherapy as well as increased the survival of treated mice (TGI = 78.1%, Fig. 8a, b). Similarly, established MC38 tumors also showed a stronger response to anti-PD-1 combination therapy than to hsBCL9_z96_ monotherapy as well as increased the survival of treated mice (TGI = 80.3%, Supplementary Fig. 14a, b). Comparable results were obtained after combined hsBCL9_z96_ and anti-CTLA-4 treatment was administered to established CT26 tumor-bearing mice (TGI = 74.8%, Supplementary Fig. 14c, d) and to established MC38 tumor-bearing mice (TGI = 78.4%, Supplementary Fig. 14e, f). In addition, anti-PD-1 therapy achieved a greater decrease in MC38 tumor growth of *Bcl9*/*Bcl9l* deficiency mice than those of *Bcl9*^f/f^*Bcl9l*^f/f^ mice as well as increased the survival in *Bcl9*^f/f^*Bcl9l*^f/f^ Cre-ERT2 mice (TGI = 92.3%, Fig. 8c, d). Together, these results indicate that therapeutic approaches aiming at overcoming immunotherapy resistance might benefit from combination with hsBCL9_z96_ to enhance antigen presentation.

## Discussion

Although immunotherapy has a promising clinical response in a small number of patients with mismatch repair deficient (dMMR) or MSI-H solid tumors, most patients with MSS or MSI-L solid tumors still do not benefit.^44,45^ To an effective antitumor response, tumor antigens have to be captured by DCs, processed into peptide fragments, and presented on DCs with MHC-I to prime CD8^+^ T cells.^1^ However, tumors exploit multiple escape mechanisms to decrease antigen presentation within TME. Amazingly, our findings uncovered that targeting BCL9/BCL9L can excellently enhance antigen presentation in cancers. We demonstrated that targeting BCL9/BCL9L facilitates the antigen presentation and tumor infiltration of cDC1, as well as boosts the tumor infiltration of CD8^+^ T cells, thereby improving the responses to cancer immunotherapy. The following aspects summarize the detailed mechanisms and importance of our findings.

First, Wnt/β-catenin signaling is recognized as a key factor to dampen tumor immune cell infiltration in many human cancers. Antigen presentation is indispensable for immune cell infiltration, especially for the tumor infiltration of CD8^+^ T cells. It was reported that inhibition of Wnt/β-catenin signaling promotes antitumor immunity by enhancing CD8^+^ T cell responses, while the mechanism of this effect is still not completely clear. cDC1 are thought to perform cross-presentation for CD8^+^ T cell priming.^3,46^ Our study demonstrated that targeting BCL9/BCL9L facilitates antigen presentation by promoting cDC1 activation and tumor infiltration. Therefore, our data may provide an excellent mechanism for inhibiting Wnt/β-catenin signaling to promotes CD8^+^ T cell-mediated antitumor responses. Second, cDC1 are often dysfunctional and absent from antigen-presenting capability for cross-priming within TME. NF-κB/IRF1 axis governs the activation and antigen presentation of cDC1.^35^ At present, the role of Wnt/β-catenin signaling in cDC1 is still elusive. Our study demonstrated that BCL9/BCL9L mediates the activation and antigen presentation of cDC1 via NF-κB/IRF1 axis. This is the first work to show that Wnt/β-catenin signaling plays a negative role in NF-κB-dependent antigen presentation of cDC1. Therefore, our study may also provide a novel mechanism for targeting Wnt/β-catenin signaling to promote antitumor immunity by overcoming cDC1 dysfunction in TME, suggesting that targeting BCL9/BCL9L to improve NF-κB-dependent antigen presentation in tumors is a potential approach to cancer therapy. Third, it is well documented that intratumoral cDC1 numbers are associated with the improved prognosis and responses to cancer immunotherapy.^9,47^ CCL4, CCL5 and XCL1, regulate migration of cDC1.^6,9,19^ We also uncovered that BCL9 and β-catenin drive Wnt signaling to promote XCR1^+^ cDC1 migration via XCL1, thereby offering a novel mechanism for targeting Wnt/β-catenin signaling to promote cDC1 tumor infiltration. Finally, DCs have been found to increase the production of CXCL9 and CXCL10 in an IFN-γ-dependent manner, which facilitates the infiltration of CD8^+^ T cells into tumors.^43^ Consistently, we also demonstrated that cDC1 in the TME produced more CXCL9 to recruit effector CD8^+^ T cells into TME through CXCL9-CXCR3 axis after inhibition of BCL9, supporting that targeting Wnt/β-catenin signaling promotes the tumor infiltration of CD8^+^ T cells.

Admittedly, manipulation of cellular microenvironment is crucial for analyzing the roles of various immune cells in checkpoint blockade outcome evaluation and prognosis prediction. In addition, we used both pharmacological inhibition and genetic depletion on the same target in our study. The key findings and mechanisms were all confirmed by these two approaches, credibly illustrating that targeting BCL9/BCL9L facilitates antitumor immunity by enhancing antigen presentation. We observed metabolic pathway changes in the tumor microenvironment which are a result of both intrinsic tumor cell characteristics and interactions with the surrounding stromal and immune cells. It appears that DC overaction may also be regulated by additional pathways rather than directly modulated by BCL9/9L targeting chemicals alone. The metabolic changes in cDC1 are interesting and further study is required to reveal the underlying mechanisms.

In the current study, we reveal a novel mechanism of positive feedback loop. Our findings indicate that targeting BCL9/BCL9L facilitates the antigen presentation and infiltration of cDC1, eliciting robust antitumor CD8^+^ T responses, further advancing our understanding of targeting Wnt/β-catenin pathway to promote antitumor immunity. Our work also provides a potential combination therapy of hsBCL9_z96_ and immune checkpoint blockade for cancer immunotherapy. However, unintended side effects should be carefully and systematically evaluated. In addition, these applications in other cancer types with survival related to *BCL9*, such as hepatocellular carcinoma, melanoma and triple negative breast cancer, need to be investigated.

In summary, our data uncover that targeting BCL9/BCL9L plays a key role in cDC1-regulated presentation of tumor-derived antigens and subsequently in cDC1-triggered activation and tumor infiltration of CD8^+^ T cells, constituting a positive feedback loop required for optimal antitumor immunity. Importantly, our study provides an excellent mechanism for inhibiting Wnt/β-catenin signaling to facilitate CD8^+^ T cell-mediated antitumor responses by promoting antigen presentation. Thus, our findings offer some insights into enhancing the susceptibility of tumors with high *BCL9*/*BCL9L* expression to cancer immunotherapy.

## Materials and methods

Further details on the experimental methods are available in the Supplementary Materials section online, under Materials and Methods.

### Mice

Female C57BL/6 J and BALB/c mice, aged 6-8 weeks, were acquired from Charles River Experimental Animal Company located in Zhejiang, China. *Bcl9*^f/f^*Bcl9l*^f/f^ Cre-ERT2 mice were obtained from Prof. Basler’s lab in Switzerland. OT-I mice were purchased from the Southern Model Animal Center (Shanghai, China). The mice were kept in a facility free from specific pathogens. Primers used for identifying the mice are detailed in Supplementary Table 1. All procedures involving animals were conducted following the approved protocols by the Animal Care and Use Committee at the School of Pharmacy, Fudan University.

### Cell lines

Human embryonic kidney 293T, human colon cancer Colo320DM and HCT116, murine colon cancer MC38 and CT26, murine melanoma B16F10, and murine breast cancer 4T1 cell lines were obtained from the American Type Culture Collection (ATCC) located in Manassas, VA, USA. Murine MC38-OVA and B16-OVA tumor cells were kindly provided by Prof. Zhijian Cai (Zhejiang University School of Medicine, Hangzhou, China). 293 T, Colo320DM, MC38, MC38-OVA, B16F10 and B16-OVA tumor cells were cultured in DMEM (SH30243.01, Hyclone) supplemented with 10% fetal bovine serum (FBS; 10099, Gibco); HCT116, CT26 and 4T1 tumor cells were cultured in RPMI 1640 (SH30809.01B, Hyclone) supplemented with 10% FBS. All these cells were incubated in standard culture conditions (37 °C in 5% CO_2_). The differentiation of CD103^+^ cDC1 in vitro was derived from primary bone marrow cells according to the induced CD103^+^ DC protocol.^48^ DCs were collected at 10–14 days for subsequent experiments.

### Tumor models and treatments

Each mouse received a subcutaneous injection 2 × 10^6^ MC38, 2 × 10^6^ MC38-OVA, 4 × 10^5^ CT26, 3 × 10^5^ B16F10, 3 × 10^5^ B16-OVA, and 3 × 10^5^ 4T1 tumor cells on the right flank. For hsBCL9_z96_ treatment, mice-bearing tumors were randomly assigned to different groups and subsequently injected intraperitoneally (i.p.) with hsBCL9_z96_ (30 mg/kg or 40 mg/kg) every day for 2 weeks, once tumor volumes reached 20-40 mm^3^. Tumor volumes and body weights of the mice were recorded every two days. Tumor volumes were determined using the formula: volume = (length × width^2^)/2. Tumor growth inhibition (TGI) index was calculated using the equation: TGI = [1−(*T*_f_/*T*_i_)/(*C*_f_/*C*_i_)] × 100%, where *T*_f_ and *C*_f_ denote the final tumor volumes of the treatment and control groups, respectively, and *T*_i_ and *C*_i_ denote the initial tumor volumes of the treatment and control groups, respectively. For CD4 and CD8 T cells depletion, individual mice were injected *i.p*. with anti-mouse CD4 monoclonal antibodies (Abs) (100 μg/100 μL; BE0003-1, BioXcell) or anti-mouse CD8 Abs (100 μg/100 μL; BE0004-1, BioXcell) on days +2, +4, +6, +8 and +10 after inoculation. For anti-PD-1 or anti-CTLA-4 combination therapy, individual mice were injected *i.p*. with anti-PD-1 (10 mg/kg; BE0146, BioXcell) or anti-CTLA-4 (10 mg/kg; BE0131, BioXcell) every 3 days. To silence *Bcl9* and *Bcl9l, Bcl9*^f/f^*Bcl9l*^f/f^ Cre-ERT2 mice received intraperitoneal injections of tamoxifen (1 mg/mouse; 105-40-29-1, Sigma) in olive oil on days −7, −6, −5, +1, +6, and +11 post inoculation.

### Treatment of tumor tissues

Tumors were excised at the end of the experiment. The weight of the tumors was measured using a precision microscale. For further analysis, the tumors were sectioned and enzymatically broken down with collagenase IV (1 mg/mL; C5138, Sigma) and DNase I (20 μg/mL; DN25, Sigma), suspended in RPMI 1640 medium, and incubated at 37 °C for 1–2 h. The single cell suspensions from tumors were passed through a 70 μm cell strainer (352350, BD Biosciences) and washed with PBS 2 times. For intracellular staining by flow cytometry and chemokine analysis by ELISA, the single cell suspensions from tumors were cultured and stimulated with cell stimulation cocktail (including protein transport inhibitors) (00-4975-03, eBioscience) and incubated for 4-6 h at 37 °C in RPMI 1640 medium. After that the cells and the supernatants were collected. The single-cell suspensions from tumors were washed with PBS for staining by flow cytometry and the supernatants were collected for chemokine analysis by ELISA. For tumor RNA isolation, tumor tissues were extracted immediately after homogenization following the manufacturer’s instructions.

### Real-time PCR

Total RNA was isolated from cells using TRIZOL reagent (15596026, Invitrogen) and subsequently transcribed into cDNA using a reverse transcription kit (RR036A, TaKaRa), adhering to the instructions provided by the manufacturer. Real-time PCR was performed employing SYBR Premix Ex Taq (RR420, TaKaRa) and specific primers, utilizing the Applied Biosystems StepOne Plus Real-Time PCR Systems. Data analysis was carried out using the 2^−ΔΔCt^ method. The sequences of the primers are detailed in Supplementary Table 2.

### ELISA

Supernatants were harvested from single-cell suspensions of tumor tissues, cultured and stimulated with a cell stimulation cocktail (including protein transport inhibitors) (00-4975-03, eBioscience) for 4-6 h at 37 °C in RPMI 1640 medium, followed by centrifugation at 2000 rpm for 10 min for chemokine analysis using ELISA kits (Mouse CXCL9/MIG ELISA Kit, 70-EK2143/2-48, MULTI SCIENCE; Mouse IFN-gamma ELISA Kit, 70-EK280/3-96, MULTI SCIENCE; Mouse lymphotactin/XCL1 ELISA Kit, SEK50677, Sino Biological) according to the manufacturer’s instructions.

### Flow cytometry

For surface staining, cells were directly stained with surface markers according to the manufacturer’s instructions. For intracellular staining, cells were first cultured and stimulated with cell stimulation cocktail (plus protein transport inhibitors) (00-4975-03, eBioscience). Following surface marker staining, the cells were fixed and permeabilized using intracellular fixation & permeabilization buffer (88-8824-00, eBioscience) following the manufacturer’s instructions. For transcription factor staining, the cells underwent a similar fixation and permeabilization process using Foxp3/Transcription factor staining buffer (00-5523-00, eBioscience) as outlined by the manufacturer, before being stained for specific transcription factors. Definitions of cell types were as follows: CD8^+^ T cells were identified as CD45^+^ CD3^+^ CD8^+^ or CD45^+^ CD8^+^ T cells, NK cells as CD45^+^ CD3^−^ NK1.1^+^ cells, and cDC1 as CD45^+^ CD11b^−^ CD11c^+^ MHC^−^II^+^ CD103^+^ cells. The specific monoclonal antibodies used are detailed in Supplementary Table 3.

### DC generation

To generate murine iCD103 DCs, bone marrow mononuclear cells after depletion of red cells were cultured in 10 mL volume of RPMI 1640 medium enriched with 10% heat-inactivated FBS (10099, Gibco), 40 ng/mL recombinant murine FLT3L (250-31 L, Peprotech) and 20 ng/mL recombinant murine GM-CSF (315-03, Peprotech) for 7 days, and subsequently replaced with the same combination of cytokines and collected at days 10 to 14 for subsequent experiments.

### Dendritic cells chemotaxis assay

The chemotaxis of cDC1 cells was evaluated using transwell migration assays. A suspension of 5×10^5^ DCs in 100 µL of RPMI 1640 medium with 2% FBS was introduced into 5 µm pore-sized transwell inserts (3421, Corning). These inserts were then placed into 24-well culture plates, each well containing 500 µL of RPMI 1640 with 10% FBS, 300 ng/mL of XCL1 (50677-M08B, Sino Biological). Following 3-h incubation at 37 °C, cells that migrated to the lower compartment were collected and quantified using flow cytometry. Migration index is calculated as = (Final lower compartment cells number/input number) _treatment group_/Mean (Final lower compartment cells number/input number) _control group_ × 100%.

### CD8^+^ T cell chemotaxis assay

Chemotaxis of CD8^+^ T cells was performed in transwell migration assays. CD8^+^ T cells were isolated from female mice using EasySep™ Mouse CD8^+^ T Cell Isolation Kit (19853, STEMCELL). They were cultured in RPMI 1640 with 10% heat-inactivated FBS, 40 U/mL recombinant murine IL-2(212-12, Peprotech) for a duration of 3 days. Afterwards, the CD8^+^ T cells were collected and washed with PBS. The cells (2×10^5^) in 100 μL were taken up in RPMI 1640 with 2% FBS and placed into 5 μm pore-sized transwell inserts (3421, Corning), which were placed into 24 wells of culture plate containing 500 μL RPMI 1640 with 10% FBS and corresponding experimental factors (recombinant murine MIG (CXCL9), 250-18, Peprotech; mouse CXCL9/MIG polyclonal Abs, AF-492-SP, R&D Systems). Following 4-h incubation at 37 °C, cells that migrated to the lower compartment of the setup were collected and their numbers determined using flow cytometry. Migration index is calculated as = (Final lower compartment cells number/input number) _treatment group_/Mean (Final lower compartment cells number/input number) _control group_ × 100%.

### CD8^+^ T cell proliferation assay in vivo

For hsBCL9_z96_ treatment model, individual mice were injected subcutaneously with 2×10^6^ MC38-OVA tumor cells on the right flank at day 0. Naïve CD8^+^ T cells purified from OT-1 TCR transgenic mice using EasySep™ Mouse Naïve CD8^+^ T Cell Isolation Kit (19858, STEMCELL). They were labeled with CFSE (5 μM; C34554, Invitrogen) and intravenously transferred (5 × 10^5^ cells/mouse) into MC38-OVA tumor-bearing mice at day 4. hsBCL9_z96_ treatment (40 mg/kg for i.p daily) was performed 12 h later for 3 days. At day 8, the mouse TdLNs were collected and subsequently analyzed using flow cytometry. For knock out mice model, *Bcl9*^f/f^*Bcl9l*^f/f^ Cre-ERT2 mice and *Bcl9*^f/f^*Bcl9l*^f/f^ mice were injected subcutaneously with 2×10^6^ MC38-OVA tumor cells on the right flank at day 0. *Bcl9*^f/f^*Bcl9l*^f/f^ Cre-ERT2 mice and *Bcl9*^f/f^*Bcl9l*^f/f^ mice were treated *i.p*. with tamoxifen (1 mg/mouse;105-40-29-1, Sigma) in olive oil on days −7, −6, −5, +1, and +6 post inoculation. Naïve CD8^+^ T cells isolated from OT-1 TCR transgenic mice were labeled with 5 μM CFSE and intravenously transferred (5×10^5^ cells/mouse) into MC38-OVA tumor-bearing *Bcl9*^f/f^*Bcl9l*^f/f^ Cre-ERT2 mice and *Bcl9*^f/f^*Bcl9l*^f/f^ mice at day 4. At day 8, the mouse TdLNs collected and were analyzed by flow cytometry.

### RNA-seq analysis

Tumor RNA was extracted and reverse transcribed into cDNA-to-cDNA libraries, followed by sequencing by TIANGEN BIOTECH (Beijing, China) using Illumina Hiseq platform. Raw reads for each sample were aligned to the mouse reference genome (GRCm38) using Hisat2 v2.0.5. The expression of individual gene was normalized to fragments per kilobase of transcript per million mapped reads (FPKM), factoring in the gene’s length and the mapped read counts. We identified significantly differentially expressed genes (DEGs) when they exhibited a fold change greater than 2 (an absolute value of Log2 ratio exceeding 1) and an adjusted p-value under 0.05, employing the DESeq2 analytical approach. For the DEGs, Gene Ontology (GO) enrichment was analyzed using the topGO package in R, considering GO terms with p-values less than 0.05 as significantly impacted. Additionally, the extent of DEG enrichment was assessed through the Kyoto Encyclopedia of Genes and Genomes (KEGG) pathways using the clusterProfiler package in R.

### Preparation of cell suspensions for single-cell RNA sequencing

The *Bcl9*^f/f^*Bcl9l*^f/f^ Cre-ERT2 mice and *Bcl9*^f/f^*Bcl9l*^f/f^ mice (6-8 weeks old) were injected subcutaneously with 3 × 10^5^ B16-OVA tumor cells on the right flank at day 0. The *Bcl9*^f/f^*Bcl9l*^f/f^ Cre-ERT2 mice and *Bcl9*^f/f^*Bcl9l*^f/f^ mice were treated *i.p*. with tamoxifen (1 mg/mouse; 105-40-29-1, Sigma) in olive oil on days −7, −6, −5, +1, +6, and +11 post inoculation. Tumors and TdLNs were collected after 3 weeks. Single-cell suspensions were obtained from tumors and TdLNs after digestion with 2 mg/mL collagenase P (Roche) and 0.2 mg/mL DNase I (Roche) at 37 °C for 15 min, followed by passing through a 40 μm cell strainer (352340, BD Biosciences).

### 10× library preparation and sequencing

10× library preparations created from single cells carrying distinct barcodes were pooled for sequencing on a Chromium Single-Cell Platform (10×Genomics Chromium^TM^) using 10× Genomics Single Cell 3′ Reagent Kits v2 (10× Genomics Chromium^TM^) following the guidelines provided by the manufacturer. The quality of the library was evaluated using a Qubit fluorometer. Sample clustering was carried out on a cBot Cluster Generation System using the TruSeq PE Cluster Kit v3. The final library was sequenced with 150 base pair paired-end reads on an Illumina HiSeq 2000.

### PCA analysis and linear dimension reduction

Genes that were detected in more than 3 cells and cells that had detective genes ranging from 200 to 2500 and the percentage of all the counts belonging to mitochondrial gene lower than 5% were kept to form high-quality data. Data were normalized by LogNormalize, an algorithm of Package Seurat before further analysis. Principle component analysis (PCA) calculates the similarity and variability of cells. For visualization, the Barnes-Hut t-Distributed Stochastic Neighbor embedding (t-SNE) was performed to reduce the dimension. The analysis above was conducted using the functions sourcing from Package Seurat version 4.0.3.

### Statistical analysis

Statistical analyses were conducted using GraphPad Prism 8.0 software. Data are presented as mean ± standard deviation (SD). To compare differences between two groups, an unpaired Student’s *t* test was utilized. Differences among multiple groups were analyzed using either one-way or two-way ANOVA, depending on the data structure. Survival rates were evaluated with the log-rank test. Pearson and Spearman Correlation Coefficients were employed to assess the relationships between genes and signaling pathways. A p-value of less than 0.05 was considered statistically significant.

### Supplementary information

SupplementaryMaterials

Author_Checklist_for_Research_Article

## Data Availability

Public Data Resources: The TCGA datasets, including COAD, SKCM, and BRCA, were obtained from GDC (https://portal.gdc.cancer.gov/). Normal skin tissue data from GTEx were sourced from UCSC Xena, available at https://xenabrowser.net/datapages/. scRNA-seq is available at: 10.6084/m9.figshare.25622307. RNA-seq is available at: https://figshare.com/s/164832440aa00ee8fc99. The complete dataset for this study, including high-throughput sequencing, flow cytometry, and immunohistochemical data, has been uploaded and is accessible at https://figshare.com/s/970ed37d44f9ac3489db.
