# Supplementary material for: Targeting BCL9/BCL9L enhances antigen presentation by promoting conventional type 1 dendritic cell (cDC1) activation and tumor infiltration
Source: Signal Transduct Target Ther. 2024 May 29;9:139. doi: 10.1038/s41392-024-01838-9 (PMC11137111; doi:10.1038/s41392-024-01838-9)
Supplement: Supplementary file 1 — SupplementaryMaterials [file 41392_2024_1838_MOESM1_ESM.docx]

**Supplementary Materials for**

**Targeting BCL9/BCL9L enhances antigen presentation by promoting conventional type 1 dendritic cell activation and tumor infiltration**

**Fenglian He,^1^ Zhongen Wu,^1^ Chenglong Liu,^1^ Yuanyuan Zhu,^1^ Yan Zhou,^2^ Enming Tian,^1^ Rina Rosin-Arbesfeld,^3^ Dehua Yang,^2^ Ming-Wei Wang,^4,6,7,8^ Di Zhu^4, 5^**

^1^Department of Pharmacology, Minhang Hospital, and Key Laboratory of Smart Drug Delivery, Shanghai Engineering Research Center of Immune Therapy, School of Pharmacy, Fudan University, Shanghai 201203, China. ^2^The National Center for Drug Screening and the CAS Key Laboratory of Receptor Research, Shanghai Institute of Materia Medica, Chinese Academy of Sciences (CAS), Shanghai 201203, China. ^3^Department of Microbiology and Immunology, Sackler Faculty of Medicine, Tel Aviv University, Tel Aviv, Israel. ^4^Department of Pharmacology, School of Basic Medical Sciences, Fudan University, Shanghai 200032, China. ^5^ Shandong Academy of Pharmaceutical Science, Jinan, China; ^6^ Research Center for Deepsea Bioresources, Sanya, Hainan 572025, China; ^7^ Department of Chemistry, School of Science, The University of Tokyo, Tokyo113-0033, Japan; ^8^ Engineering Research Center of Tropical Medicine Innovation and Transformation of Ministry of Education, School of Pharmacy, Hainan Medical University, Haikou 570228, China;

Correspondence to: mwwang@simm.ac.cn or zhudi@fudan.edu.cn

**This file includes:**

Materials and Methods

Figures. S1 to S14

Tables S1 to S3

Reference

# Materials and Methods

## Cell proliferation assay

Cell proliferation of Colo320DM was measured with BrdU ELISA kit (11647229001, Roche) following the protocol provided by the manufacturer, which aligns with the methods outlined in our earlier research ^1^. We measured absorbance using a microplate reader at 450 nm, using 690 nm as the reference wavelength.

## LEF/TCF reporter assay

We quantified reporter activity using the CellSensor® LEF/TCF-bla HCT116 (K1178, Invitrogen). The dose-dependent inhibition curves of hsBCL9_z96_ (TFA salts) in the Wnt/β-catenin signaling pathway were evaluated with this system, employing protocols established in our earlier studies.^1^

## Toxicity study

Histopathological analysis was used to assess drug toxicity. Organs including the heart, liver, spleen, lung, kidney, and small intestine were collected from CT26 tumor-bearing mice treated with either vehicle control or hsBCL9_z96_ at 30 mg/kg intraperitoneally, daily for 14 days. Hematoxylin and eosin (H&E) staining was performed on these samples following the manufacturer’s instructions.

## Multiplex immunofluorescence

The multiplexed analysis of cDC1 (marked CD103^+^ CD11c^+^) was performed on the 4% paraformaldehyde-fixed and paraffin embedded of TdLNs from different murine models by Wisee Biotechnology (Shanghai, China). The sections were stained for anti-CD103(ab254182, abcam), anti-IRF1(8478, Cell Signaling Technology), anti-p-TAK1 (bs-3439R, Bioss) and anti-CD11c (97585S, Cell Signaling Technology) by using 4-color Novo-Light™ multiplex fluorescence immunohistochemistry. Briefly, The sections were incubated with primary antibodies for 60 minutes. including CD103, IRF1, p-TAK1 and 30 minutes with CD11c antibody at room temperature, and then treated with poly-HRP anti-mouse/rabbit IgG for 10 minutes, followed by incubation with the corresponding 4-color fluorophores for an additional 10 minutes(Neon TSA520, Neon TSA570, Neon TSA670 and Neon TSA620). Nuclei was counterstained with DAPI (Blue). The whole sections were scanned using a confocal laser scanning microscope. Representative cells from the single-color slides were then analyzed with CaseViewer software 2.4 , provided by 3DHISTECH Ltd. in Budapest, Hungary.

## Biacore assay

β-catenin was immobilized on a CM5 chip. Sensogram was obtained by using a series of 2-fold diluted concentrations of hsBCL9_z96_ with 62.5 nM as the highest concentration. The flow rate was 30 µl/min. Both the association and dissociation times were set at 120 seconds. The 1:1 binding model was used for evaluating the binding of peptide hsBCL9_z96_ to β-catenin. The K_D_ value was evaluated using Biacore T200 software.

## HPLC/MS

Seqeuence of hsBCL9_Z96_: Ac– Leu-Gln-Thr-Leu-Arg-S5-Ile-Gln-Arg-S5-Leu-2Nal- betaAla-betaAla-Gly-Arg-Lys-Lys-Arg-Arg-Gln-Arg- Arg-Arg-Pro-Gln-Lys(PEG4-palmitoyl)-NH2. Purity by HPLC:95.78% (214 nm), RT= 15.58 min, Mobile Phase: A: 0.05% TFA in water, B: 0.05% TFA in ACN, Gradient: 20%B for 1 min, 20-80% B within 20 min, Flow Rate: 1.0 mL/min, Column Temperature: 40 ℃, Column: XBridge Peptide BEH C18, 4.6* 150mm, 3.5 μm, LC/MS:(ESI) m/z: 798.7 [M+5H]/5+,666.0 [M+6H]/6+，95.78% (214 nm), RT= 15.58 min, Mobile Phase: A: 0.05% TFA in water, B: 0.05% TFA in ACN, Gradient: 20% B for 1 min, 20-80% B within 20 min, Flow Rate: 1.0 mL/min, Column Temperature: 40℃, Column: XBridge Peptide BEH *C18, 4.6* 150mm, 3.5 μm* (supplementary Fig. 3k and 3l)

## Bioinformatics analysis of TCGA data

The transcriptome expression profiles, measured as FPKM (Fragments Per Kilobase of transcript per Million mapped reads), and the corresponding clinical information of colon adenocarcinoma (COAD), skin cutaneous melanoma (SKCM) and breast invasive carcinoma (BRCA) from The TCGA datasets were down loaded from GDC (https://portal.gdc.cancer.gov/). The FPKM of normal skin tissue from GTEx were down loaded from UCSC xena (https://xenabrowser.net/datapages/). TCGA COAD data were stratified to MSS, MSI-L and MSI-H according to a previous study^2^. For analysis of gene signature, HLA-I signature, Antigen processing and presentation signature, *Ifng* signature and CD8 effector T cells signature were previously defined. In detail, HLA-I signature includes the following genes: *TAP1,* *HLA-B,* *PSMB8 (LMP7), HLA-F, HLA-A, PSMB9 (LMP2), HLA-H, HLA-C, HLA-E, HLA-L, HLA-K, HLA-G, HLA-J, TAP2*^3^*.*Antigen processing and presentation signature includes the following genes: *HSPA1A, HSPA1B, CD74, CTSS, HLA-B, HLA-A, HLA-C, HSPA4, CD8A, CD1D, HLA-DRB1, HLA-DQA1, HLA-DQB1, IFNA1, IFNA2, HLA-DMB, CD1A, CD1B, HLA-DPA1, HLA-DPB1, HSPA5, HLA-DRA, HLA-DRB5, IFNG, HSPA6, HSPA8, HSPA1L, HSPA2, ICAM1, FCGRT, FCER1G, KIR2DL1, KIR2DL3, KIR2DL4, KIR3DL1, KIR3DL2, CD1C, CD1E, CD8B, HLA-DOA, HLA-DOB, KLRC1, KLRC2, KLRC3, KLRC4, KLRD1, CD209, HLA-DQA2, CALR, CANX, HFE, MR1, CREB1, HLA-E, HLA-F, HLA-G, CTSB, CTSE, NFYA, NFYB, NFYC, CIITA, MICA, MICB, HLA-DMA, LTA, B2M, PDIA3, AZGP1, HSP90AA1, HSP90AB1, IFNA4, IFNA5, IFNA6, IFNA7, IFNA8, IFNA10, IFNA13, IFNA14, IFNA16, IFNA17, IFNA21, KIR3DL3, RAET1E, RAET1L, UBR1, RAET1G, ULBP3, ULBP2, ULBP1, TAP1, TAP2, TAPBP, PSMC1, PSMC2, PSMC3, PSMC4, PSMC5, PSMC6, PSMB8, PSMD1, PSMD2, PSMD3, PSMD4, PSMD5, PSMD6, PSMD7, PSMD8, PSMD10, PSMD11, PSMD13, PSMD14, PSME1, PSME2, PSME3, RFX5, RFXAP, RFXANK, SLC10A2, THBS1, AP3B1, ADRM1, KIAA0368, TRPC4AP, UBXN1, ERAP1, TAPBPL, ERAP2, IFI30, PROCR, CD4, LGMN*^4^*.* *Ifng* signature includes the following genes: *IDO1, CXCL10, CXCL9, HLA-DRA, STAT1* and *IFNG*^5^*.* CD8 effector T cells signature includes the following genes: *IFNG, PRF1, CXCL10, CXCL9, GZMB, GZMA, CD8A TBX2*^6^.The information of gene sets REACTOME_TAK1_ACTIVATES_NFKB_BY_PHOSPHORYLATION_AND_ACTIVATION_OF_IKKS_COMPLEX and REACTOME_TRAF6_MEDIATED_INDUCTION_OF_TAK1_COMPLEX_WITHIN_TLR4_COMPLEX, were downloaded from Molecular Signatures Database v7.4^7^. cDC1 gene set contains *Xcr1* and *Clec9a*^8^*.* We used GSEA in R package to calculate signature score for each sample^3^. The GSVA score was calculated using gsva function from Package GSVA version 1.40.1. Immune scores can be determined using the ESTIMATE algorithm, which analyzes the gene expression profiles of immune and stromal cells in each patient to estimate immune infiltration ^9^. TCGA COAD data were stratified to without or with Wnt/β-catenin signaling activation according to a study by Sanchez-Vega et al^10^. For analysis of CD8^+^ T cells immune infiltration, we used the CIBERSORT with an expression-based deconvolution algorithm (https://cibersortx.stanford.edu/) to estimate the abundance of 22 types of immune cells in tumors as previously reported^11^. Overall Survival analysis were performed by ggsurvplot function of survminer package with a function of survcutpoint that can directly calculate the truncation value of continuous variables of survival data. Bar and violin plots were plotted with GraphPad Prism 8.0 software. Box plots and correlation dot plots were plotted with R software.

## Differential expressed gene analysis and GO enrichment of single-cell transcriptomics analysis

Significant differential expressed genes (DEGs) were defined as log2 (fold change) >0.75 (fold change >1.68 times) with adjusted *p*-value of <0.05 between the groups using FindMarkers function in Package Seurat version 4.0.3. Gene Ontology (GO) enrichment analysis of differentially expressed genes (DEGs) was conducted using the clusterProfiler R package. GO terms with a corrected p-value below 0.05 were deemed significantly enriched by DEGs.

**GSVA**

Package GSVA version 1.40.1 are used to perform GSVA^12^. For cDC1, T cells and tumor cells, normalized expression data and phenotype information describing their *Bcl9/Bcl9l* status were prepared. The information of gene sets, C2: KEGG subset of Canonical Pathways, C2: REACTOME subset of Canonical Pathways and C5: GO BP, were downloaded from Molecular Signatures Database v7.4 and used to perform GSVA and calculate the GSVA score of each pathway in each cell^7^. To analyzing the difference between cells from B16-OVA tumor-bearing *Bcl9*^f/f^*Bcl9l*^f/f^ Cre-ERT2 mice and *Bcl9*^f/f^*Bcl9l*^f/f^ mice treated i.p. with tamoxifen (1 mg/100 μL) in olive oil on days -7, -6, -5, +1, +6 and +11 post inoculation, t scores are calculated by two-sided Unpaired Student’s t-test, and *p* < 0.05 is considered to be significant. Bar plots were made based on the *t* scores to presented difference between cells from B16-OVA tumor-bearing *Bcl9*^f/f^*Bcl9l*^f/f^ Cre-ERT2 mice and *Bcl9*^f/f^*Bcl9l*^f/f^ mice treated i.p. with tamoxifen (1 mg/100 μL) in olive oil on days -7, -6, -5, +1, +6 and +11 post inoculation in the pathways that we concerned and had marked p value. To analyzing the difference between *Map3k7* (*Tak1*) KO and WT DC, data was downloaded from GSE34417. GSVA score was calculated by GSVA function and scaled by sample before plotted in a heatmap.

## GSEA

For cDC1 and T cells, normalized expression matrix and phenotype information describing their *Bcl9/Bcl9l* status were input into software GSEA version 4.1.0^13^. The information of gene sets, C2: KEGG subset of Canonical Pathways, C2: REACTOME subset of Canonical Pathways and C5: GO BP, was downloaded from Molecular Signatures Database v7.4 and applied to perform GSEA^7^. Other settings were accorded to default settings of GSEA software.

## Cell interaction analysis

For cDC1, T cells and tumor cells, normalized expression data and phenotype information describing their *Bcl9/Bcl9l* status were input into program CellphoneDB v2.0^14^. This algorithm evaluates receptor-ligand interactions between two distinct cell types by analyzing the expression of a receptor in one cell type and its corresponding ligand in another. *p*<0.05 means the receptor-ligand interactions was enriched between two cell types. Bubble plots were plotted with color showing expression and size showing *p* value.

## Single-cell regulatory network inference and clustering (SCENIC) analysis

For cDC1, normalized expression matrix were input, and SCENIC analysis is performed using the 20-thousand motifs database for RcisTarget and GENIE3^15^. Functions are sourced from Package SCENIC version 1.2.4.

## Pseudo-time analysis

For cDC1, UMI matrix are input to perform pseudo-time analysis. Differential genes, filtered by using dispersionTable function, were chosen for further analysis. Dimension reduction was performed with DDRTree and pseudo-time was calculated by using orderCells function. Functions above are sourced from Package Monocle version 2.20.0^16^.

## Conversion between human genes and murine genes

Ligand-receptor interaction pairs in cellphoneDB and genes in gene sets downloaded from Molecular Signatures Database are human-specific, and therefore conversion between human genes and murine genes is needed. The document containing homologous information is downloaded from Ensembl (<https://asia.ensembl.org/index.html>).

**anti-XCL1 blockade of XCL1/XCR1 signaling.**

Individual mice were subcutaneously injected with 3×10^5^ CT26 tumor cells on the right flank. Upon reaching tumor volumes of approximately 20-40 mm^3^, tumor-bearing mice were randomized and treated intraperitoneally with 30 mg/kg of hsBCL9_z96_ daily for two weeks. To neutralize XCL1, mice were injected intraperitoneally with 50 μg of anti-XCL1 antibody (MAB486, R&D Systems) or isotype-matched control antibodies (MAB006, R&D Systems) three times a week for two weeks.

**SIINFEKL-H2Kb complexes on cDC1.**

Each mouse received a subcutaneous injection of 2×10^6^ MC38-OVA tumor cells on the right flank. For hsBCL9_z96_ treatment, tumor-bearing mice were randomized and administered 40 mg/kg of hsBCL9_z96_ intraperitoneally, once tumor volumes reached approximately 20-40 mm^3^. OVA-I presenting on cDC1 of TDLNs were analyzed by staining anti-mouse H-2K^b^ bound to SIINFEKL (141605, Biolegend) after 5 days hsBCL9_z96_ treatment. To silence *Bcl9* and *Bcl9l,* *Bcl9*^f/f^*Bcl9l*^f/f^ Cre-ERT2 mice were treated i.p. with tamoxifen (1 mg/mouse; 105-40-29-1, Sigma) in olive oil on days -7, -6, -5 and +1 post inoculation. OVA-I presenting on cDC1 of TDLNs were analyzed by staining anti-mouse H-2K^b^ bound to SIINFEKL (141605, Biolegend) at +5 day post inoculation.

**Frequency of OVA_257–264_-specific CD8+ T cells.**

Each mouse received a subcutaneous injection of 2×10^6^ MC38-OVA tumor cells on the right flank. For hsBCL9_z96_ treatment, tumor-bearing mice were randomized and injected intraperitoneally with hsBCL9_z96_ (40 mg/kg) every day for 2 weeks, once tumor volumes reached approximately 20-40 mm^3^. To silence *Bcl9* and *Bcl9l,* *Bcl9*^f/f^*Bcl9l*^f/f^ Cre-ERT2 mice were treated intraperitoneally with tamoxifen (1 mg/mouse; 105-40-29-1, Sigma) in olive oil on days -7, -6, -5, +1, +6 and +11 post inoculation. Tumors were excised at the end of the experiment. For subsequent analysis, tumors were cut into pieces and digested with Collagenase IV (1 mg/mL; C5138, Sigma) and DNase I (20 μg/mL; DN25, Sigma) in RPMI 1640 for 1-2 h at 37 °C. The single cell suspensions from tumors were passed through a 70 μm cell strainer (352350, BD Biosciences). Cells were washed washed with PBS for 2 times and stained with Clear Back (MTG-001, MBL International), T-Select H-2Kb OVA Tetramers-SIINFEKL (TS-5001-2C, MBL International), anti-CD8 (D271-5, MBL International), anti-CD45 (45-0451-82, eBioscience) anti-CD3e (11-0031-82, eBioscience) and Fixable Viability Dye eFluor™ 450 FVD (65-0863-14, eBioscience) was used to gate living cells.

**Cross-presentation *in vitro* DC-CD8^+^ T cells proliferation assay.**

Individual mice were injected subcutaneously with 2×10^6^ MC38-OVA tumor cells on the right flank. For hsBCL9_z96_ treatment, tumor-bearing mice were randomized and then administered 40 mg/kg of hsBCL9_z96_ intraperitoneally every day. At 5-7 days later, CD11c^+^ DCs were purified from the TDLNs, and co-cultured with CFSE-labeled naïve CD8^+^ T cells isolated from the spleens and LNs of OT-1 transgenic mice at a ratio of 1:3. The CFSE dilution of proliferated OT-I CD8^+^ T cells were analyzed by flow cytometry on day 3.

*Bcl9*^f/f^*Bcl9l*^f/f^ Cre-ERT2 mice and *Bcl9*^f/f^*Bcl9l*^f/f^ mice were injected subcutaneously with 2 × 10^6^ MC38-OVA tumor cells on the right flank at day 0. *Bcl9*^f/f^*Bcl9l*^f/f^ Cre-ERT2 mice and *Bcl9*^f/f^*Bcl9l*^f/f^ mice were treated i.p. with tamoxifen (1 mg/mouse ;105-40-29-1, Sigma) in olive oil on days -7, -6, -5, +1 and +6 post inoculation. At 5-7 days later, CD11c^+^ DCs were purified from the TDLNs, and co-cultured with CFSE-labeled naïve CD8^+^ T cells isolated from the spleens and LNs of OT-1 transgenic mice at a ratio of 1:3. On day 3, the proliferation of OT-I CD8^+^ T cells, indicated by CFSE dilution, was assessed using flow cytometry.

**IFN-**γ **ELISPOT assay.**

Each mouse was subcutaneously injected with 2×10^6^ MC38-OVA tumor cells on the right flank. For hsBCL9_z96_ treatment, tumor-bearing mice were randomized and then administered 40 mg/kg of hsBCL9_z96_ intraperitoneally every day. At 5-7 days later, CD11c^+^ DCs were purified from the TDLNs, and co-cultured with naïve CD8^+^ T cells isolated from the spleens and LNs of OT-1 transgenic mice at a ratio of 1:5 in an IFN-γ ELISPOT plate for 24 hr. IFN-γ producing cells were quantified using an ELISPOT assay, following the manufacturer’s instructions.

*Bcl9*^f/f^*Bcl9l*^f/f^ Cre-ERT2 mice and *Bcl9*^f/f^*Bcl9l*^f/f^ mice were injected subcutaneously with 2 × 10^6^ MC38-OVA tumor cells on the right flank at day 0. *Bcl9*^f/f^*Bcl9l*^f/f^ Cre-ERT2 mice and *Bcl9*^f/f^*Bcl9l*^f/f^ mice were treated i.p. with tamoxifen (1 mg/mouse ;105-40-29-1, Sigma) in olive oil on days -7, -6, -5, +1 and +6 post inoculation. At 5-7 days later, CD11c^+^ DCs were purified from the TDLNs, and co-cultured with naïve CD8^+^ T cells isolated from the spleens and LNs of OT-1 transgenic mice at a ratio of 1:5 in an IFN-γ ELISPOT plate for 24 hr. IFN-γ producing cells were quantified using an ELISPOT assay, following the manufacturer’s instructions.

Individual mice were injected subcutaneously with 2×10^6^ MC38-OVA tumor cells on the right flank. For hsBCL9_z96_ treatment, tumor-bearing mice were randomized and injected i.p. with 40 mg/kg hsBCL9_z96_ every day. At 10 days later, CD8^+^ T cells isolated from TDLNs and stimulated with 10 μg/ml of OVA-I peptide in an IFN-γ ELISPOT plate for 24 hr. IFN-γ producing cells were determined by ELISPOT assay according to the manufacturer’s instructions.

*Bcl9*^f/f^*Bcl9l*^f/f^ Cre-ERT2 mice and *Bcl9*^f/f^*Bcl9l*^f/f^ mice were injected subcutaneously with 2 × 10^6^ MC38-OVA tumor cells at day 0. *Bcl9*^f/f^*Bcl9l*^f/f^ Cre-ERT2 mice and *Bcl9*^f/f^*Bcl9l*^f/f^ mice were treated i.p. with tamoxifen (1 mg/mouse ;105-40-29-1, Sigma) in olive oil on days -7, -6, -5, +1 and +6 post inoculation. At 10 days later, CD8^+^ T cells isolated from TDLNs and stimulated with 10 μg/ml of OVA-I peptide for 24 hr in an IFN-γ ELISPOT assay (*n* = 3). IFN-γ producing cells were determined by ELISPOT assay according to the manufacturer’s instructions.


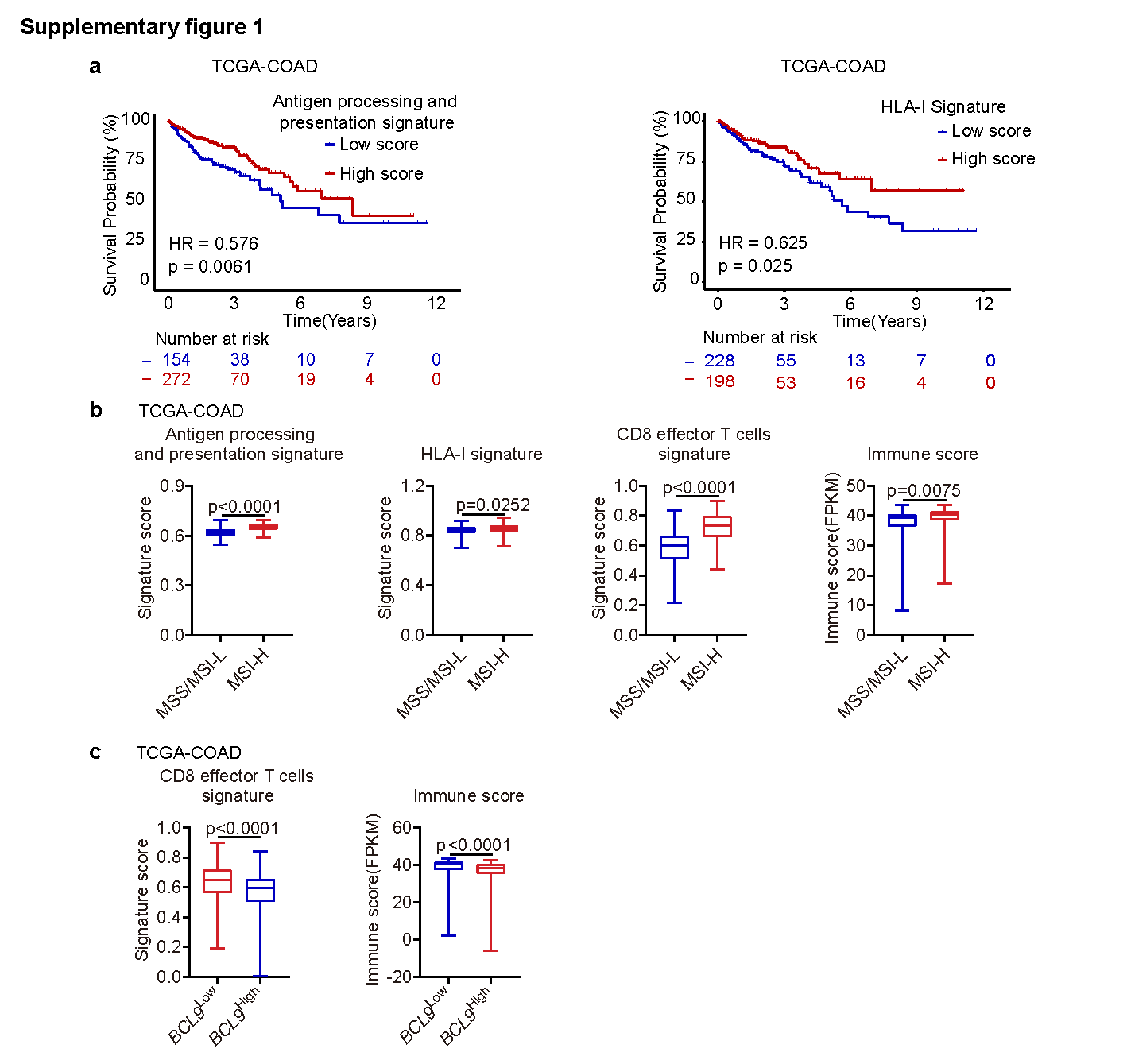


**Fig. S1. *BCL9* expression is negatively associated with** **antigen presentation in colon cancer.** (**a**) Analysis of overall survival time between low and high Antigen processing and presentation signature and HLA-I signature from TCGA COAD datasets (*n* = 426). (**b**) Analysis of Antigen processing and presentation signature, HLA-I signature, CD8 effector T cells signature and immune scores between MSS/MSI-L and MSI-H in TCGA COAD datasets (MSS/MSI-L, *n* = 277; MSI-H, *n* = 61). (**c**) Analysis of CD8 effector T cells signature and immune scores between low and high *BCL9* (median value) expression in TCGA COAD datasets (*BCL9*^Low^, *n* = 209; *BCL9*^High^, *n* = 236). These data are presented as the mean ± SD for each group; 'n' represents the number of biological replicates; statistical analyses were conducted using the Log rank test (a) and the Unpaired Student’s t-test (b and c).


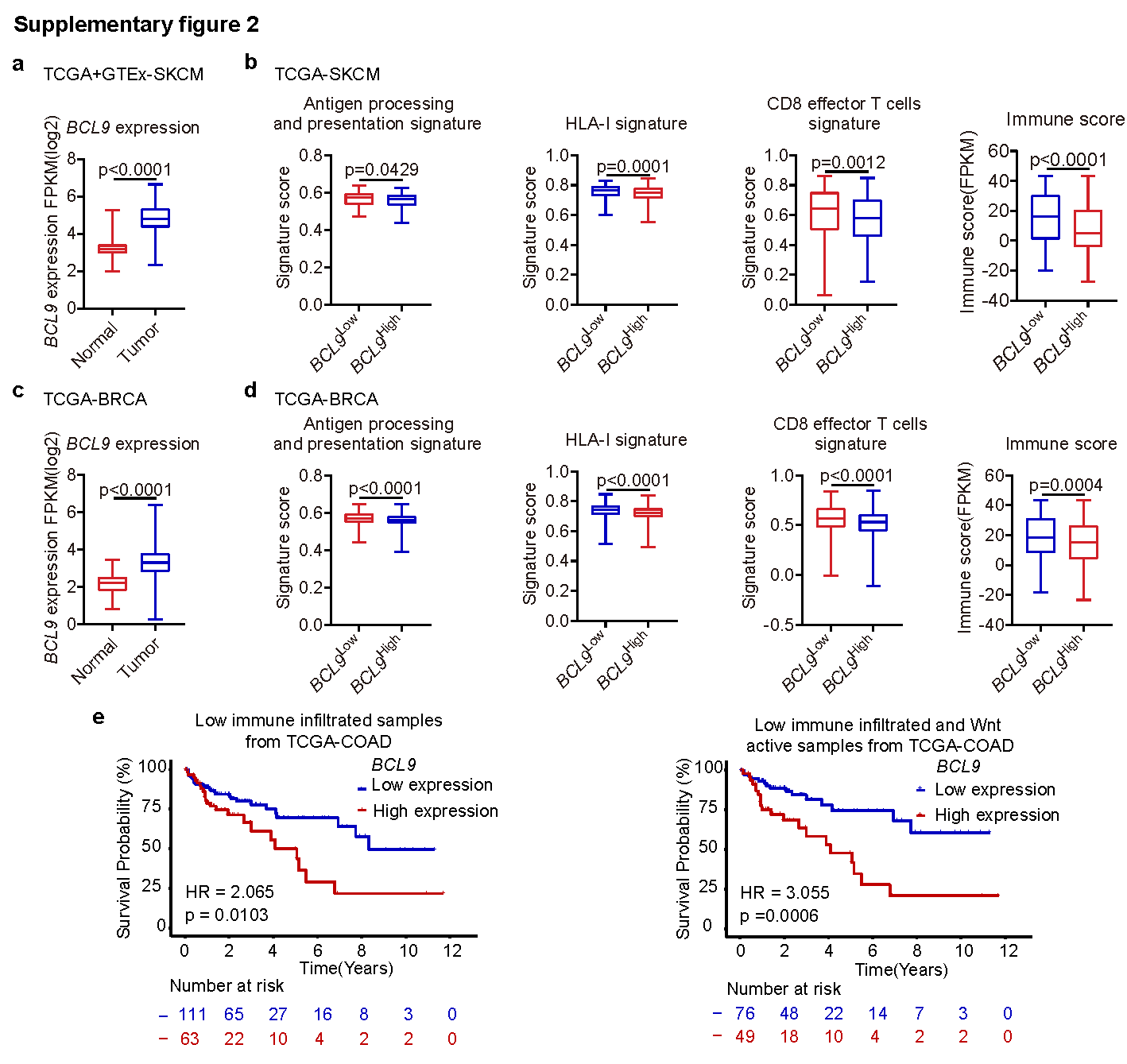


**Fig. S2. *BCL9* expression is negatively associated with** **antigen presentation in melanoma and breast cancer.** (**a**) Analysis of *BCL9* expression between tumors in TCGA SKCM datasets and normal tissues in GTEx datasets (Normal, *n* = 1810; Tumor, *n* = 470). (**b**) Analysis of Antigen processing and presentation signature, HLA-I signature, CD8 effector T cells signature and immune scores between low and high *BCL9* expression (median value) in TCGA SKCM datasets (*BCL9*^Low^, *n* = 235; *BCL9*^High^_,_ *n* = 234). (**c**) Analysis of *BCL9* expression between normal and tumor tissues in TCGA BRCA datasets (Normal, *n* = 113; Tumor, *n* = 1099). (**d**) Analysis of Antigen processing and presentation signature, HLA-I signature, CD8 effector T cells signature and immune scores between low and high *BCL9* expression (median value) in TCGA BRCA datasets (*BCL9*^Low^_,_ *n* = 493, *BCL9*^High^_,_ *n* = 604). (**e**) Analysis of overall survival time between low and high *BCL9* expression in low immune infiltrated samples and low immune infiltrated and Wnt active samples from TCGA COAD datasets (*n* = 174). The charts and values shown represent the mean ± SD for each group. 'n' denotes the number of biological replicates. Statistical analysis was performed using the Unpaired Student’s t-test (a-d) and the Log rank test (e).


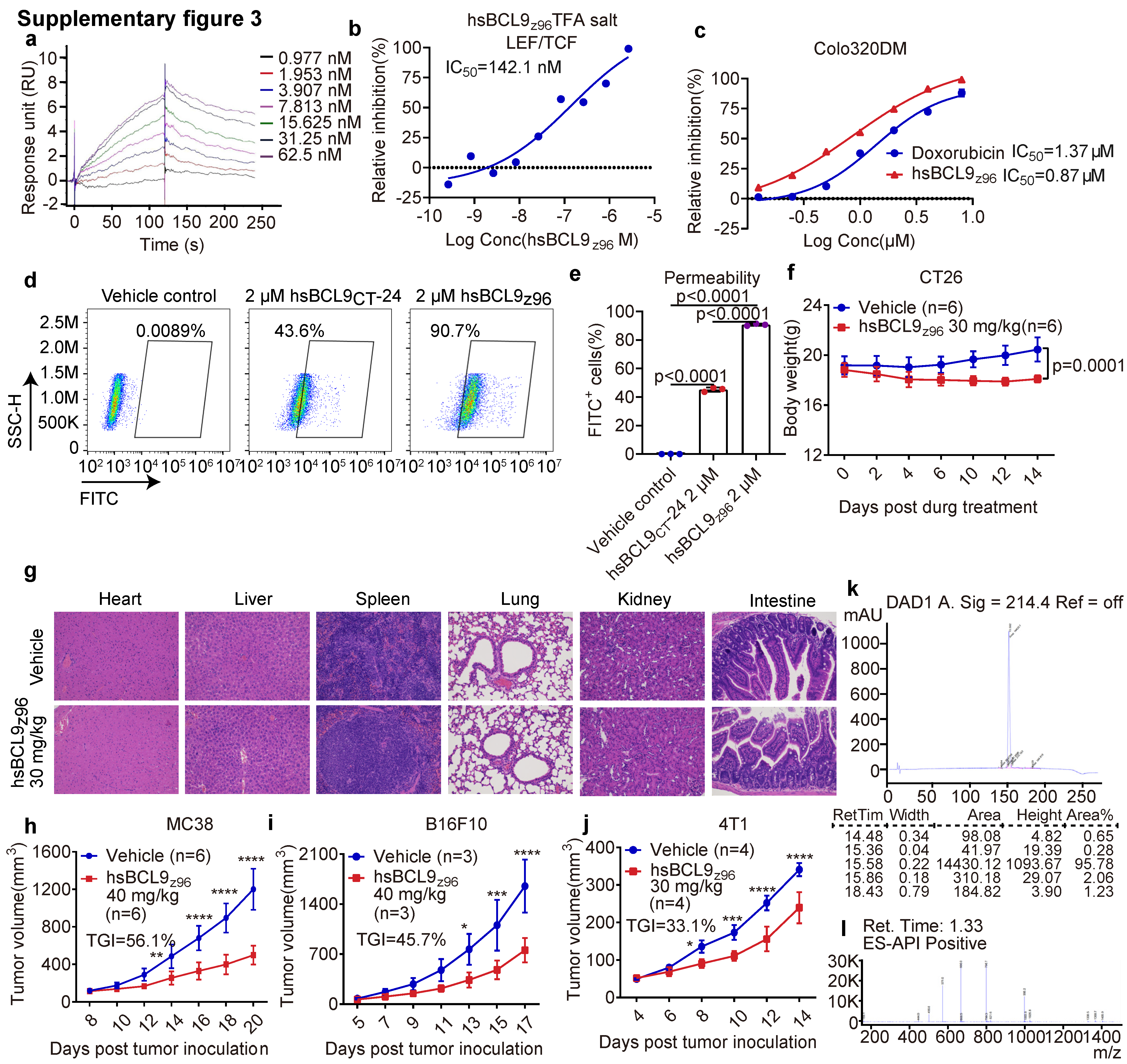


**Fig. S3. hsBCL9_z96_ demonstrates a potent profile and suppresses tumor growth.** (**a**) The β-catenin interaction with increased concentrations of hsBCL9_z96_ were measured by Biacore T200. (**b**) Dose response curves of hsBCL9_z96_ TFA salt in inhibiting the LEF/TCF pathway. (**c**) Cell proliferation of Colo320DM cells treated with hsBCL9_z96_ and doxorubicin over 24 h with the indicated doses were measured by BrdU ELISA. (**d**) Cell permeability of HCT116 cells treated with FITC-hsBCL9_z96_ and hsBCL9_CT_-24 over 24 h with the indicated doses were measured by flow cytometry. (**e**) Quantitative analysis of results of (d) (n = 3). (**f**) Body weight of hsBCL9_z96_-treated CT26 tumor-bearing mice (*n* = 6). (**g**) Representative histological appearance of the heart, liver, lung, kidney, spleen and intestine from 30 mg/kg hsBCL9_z96_-treated CT26 tumor-bearing mice. Scale bar, 200 μm (*n* = 3). (**h**-**i**) Tumor growth of hsBCL9_z96_-treated MC38 tumor-bearing mice(H)_,_ B16F10 tumor-bearing mice (I) and hsBCL9_z96_-treated 4T1 tumor-bearing mice (J) (*n* = 3-6). (**k**) HPLC of hsBCL9_Z96_ and (**l**) LC/MS of hsBCL9_Z96_. The presented data are representative values expressed as the mean ± SD of each group from three independent experiments. 'n' denotes the number of biological replicates. Significance levels are indicated as *p<0.05, **p<0.01, ***p<0.001, ****p<0.0001. Statistical analyses were performed using One-way ANOVA followed by Bonferroni test (**e**), Unpaired Student’s t-test (**f**), and Two-way ANOVA followed by Bonferroni test (**h-j**).

_
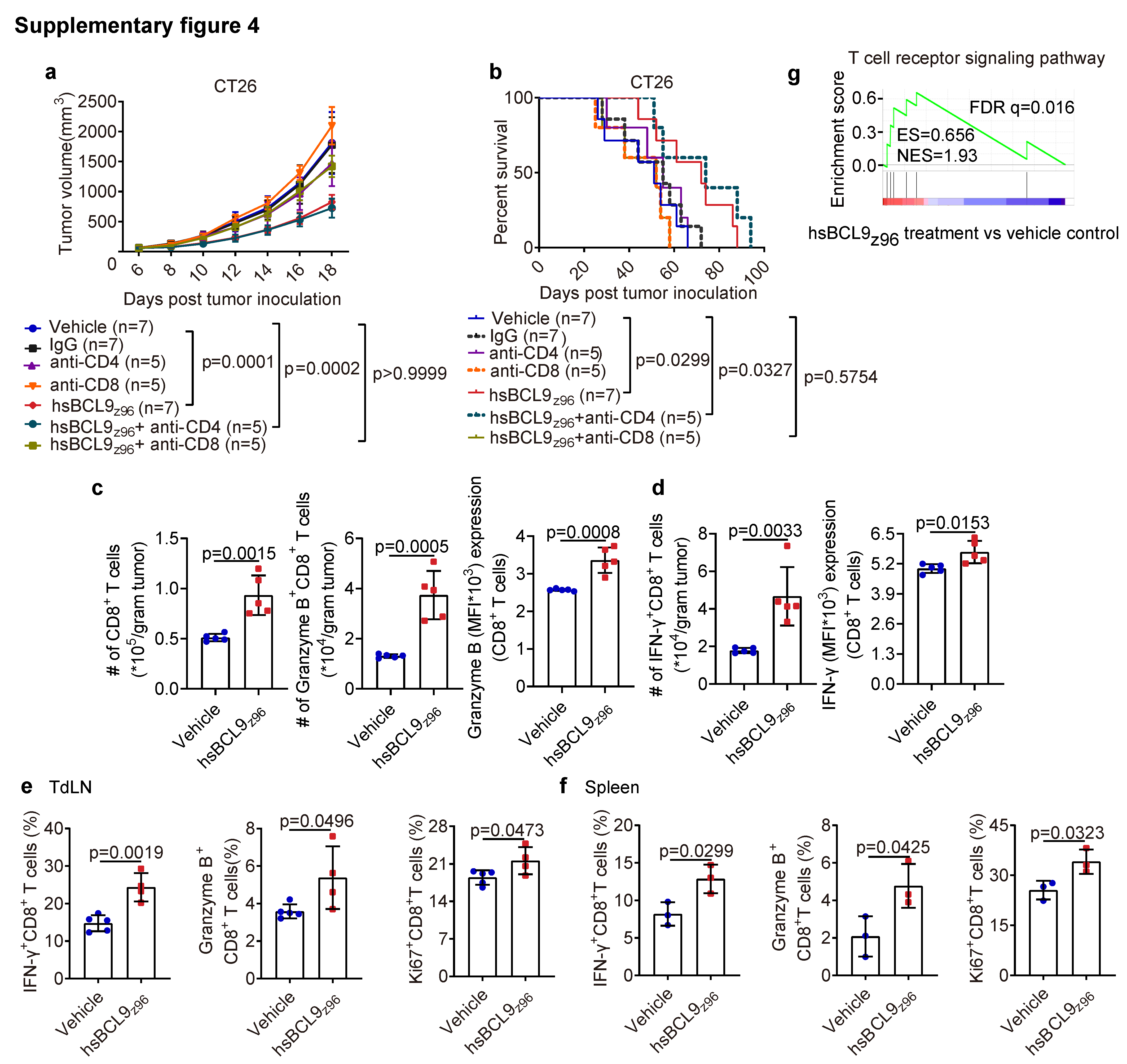
_

**Fig. S4. hsBCL9_z96_ treatment reduces tumor growth by promoting the antitumor CD8^+^ T cell responses.** (**a** and **b**) Tumor growth (a) and survival (b) of CT26 tumor-bearing mice treated with vehicle or 30 mg/kg hsBCL9_z96_. In addition, mice were injected intraperitoneally with 100 μg of either anti-CD4 or anti-CD8 antibodies on days +2, +4, +6, +8, and +10 following inoculation (n = 5-7). (**c**) IFN-γ^+^ CD8^+^, Granzyme B^+^ CD8^+^, and Ki-67^+^ CD8^+^ T cells in TILs from 30 mg/kg hsBCL9_z96_-treated CT26 tumors were analyzed by flow cytometry. (**d**) Quantitative analysis of results of (C) (*n* = 3-5). (**e** and **f**) Quantitative analysis of IFN-γ^+^ CD8^+^, Granzyme B^+^ CD8^+^, and Ki67^+^ CD8^+^ T cells in the TdLNs (e) or spleens (f) from 30 mg/kg hsBCL9_z96_-treated CT26 tumor-bearing mice were analyzed by flow cytometry (*n* = 3-5). (**g**) Upregulated GO terms of T CELL RECEPTOR SIGNALING PATHWAY in 30 mg/kg hsBCL9_z96_-treated CT26 tumors (Vehicle, *n* = 4; hsBCL9_z96_, *n* = 5). These data represent charts or values expressed as the mean ± SD of each group from three independent experiments, with n indicating biological replicates. Statistical analysis included Two-way ANOVA followed by Bonferroni test (**a**), Log rank test (**b**), and Unpaired Student’s t test **(d-f**).


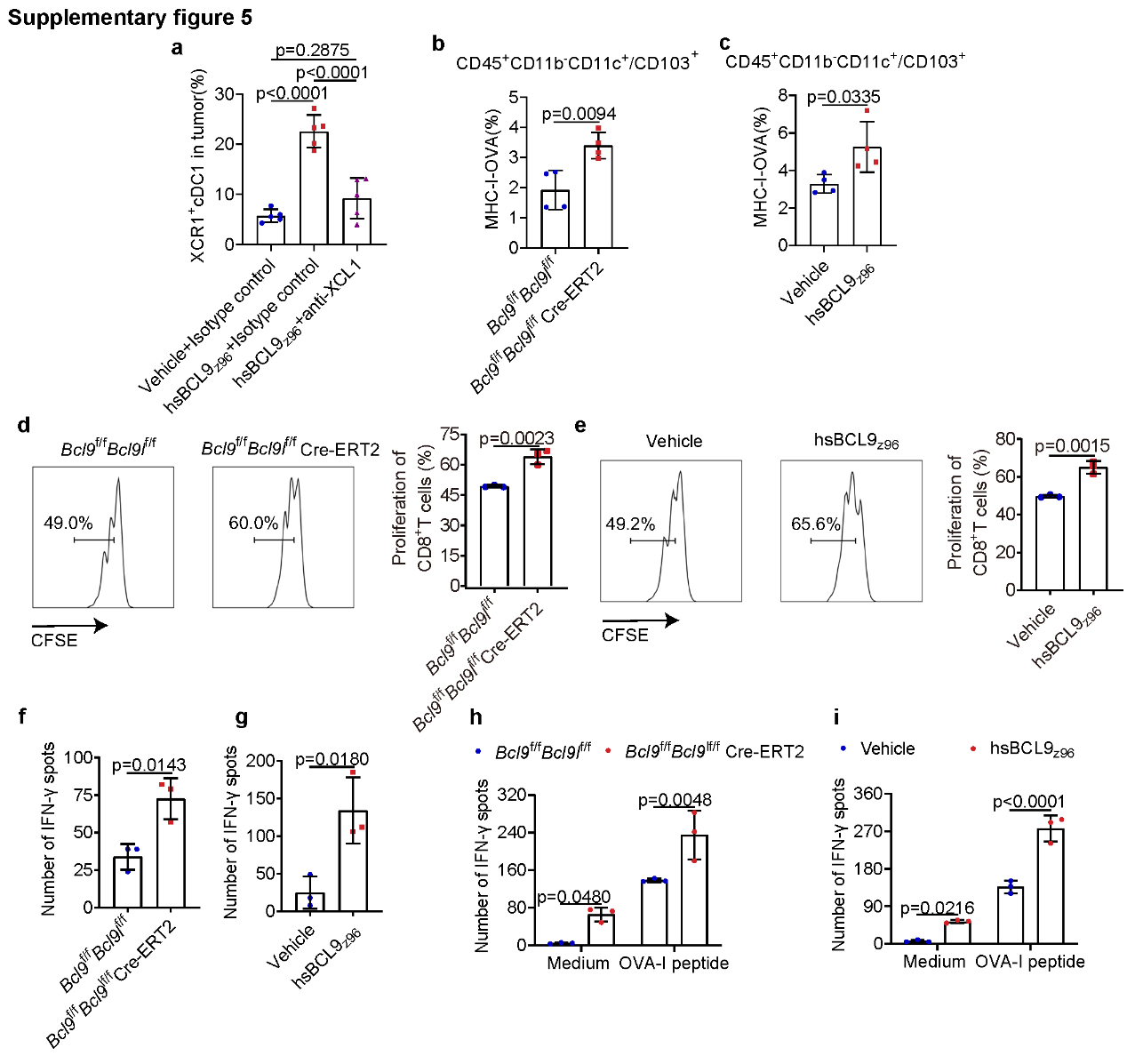


**Fig. S5. Inhibition of BCL9/BCL9L induces cDC1 activation and facilitates cross-priming of CD8^+^ T cells. (a)** The cDC1 in TILs of 30 mg/kg hsBCL9_z96_ -treated CT26 tumors from BALB/c mice injected with anti-XCL1 antibodies or the respective isotype-matched controls (*n* = 5). These data represent values expressed as the mean ± SD of each group; *n* indicates biological replicate; One-way ANOVA followed by Bonferroni test. (**b** and **c**) The analysis of OVA-I presenting on cDC1 (CD45^+^ CD11b^-^ CD11c^+^ CD103^+^) from TDLNs of hsBCL9_z96_-treated MC38-OVA tumor-bearing mice (left) or MC38-OVA tumor-bearing *Bcl9*/*Bcl9l* deficient mice (right) by flow cytometry (*n* = 4). (**d**) Representative plot (left) and quantitative analysis (right) of the proliferation of CFSE-labeled naive OT-I CD8^+^ T cells on day 3 co-cultured with CD11c^+^ DCs isolated from TDLNs of MC38-OVA tumor-bearing *Bcl9*^f/f^*Bcl9l*^f/f^ Cre-ERT2 mice treated i.p. with tamoxifen (1 mg/100 μL) in olive oil on days -7, -6, -5, +1 and +6 post inoculation at at a ratio of 3:1 were analyzed by flow cytometry (*n* = 3). (**e**) Coculture of purified CD11c^+^ DCs isolated from TDLNs of 40 mg/kg hsBCL9_z96_-treated MC38-OVA tumor-bearing mice were incubated 24 hr with naive OT-1 CD8^+^ T cells at a ratio of 1:5 in an IFN-γ ELISPOT assay (*n* = 3). (**f**) Coculture of purified CD11c^+^ DCs isolated from TDLNs of 40 mg/kg hsBCL9_z96_-treated MC38-OVA tumor-bearing mice were incubated 24 hr with naive OT-1 CD8^+^ T cells at a ratio of 1:5 in an IFN-γ ELISPOT assay (*n* = 3). (**g**) Coculture of purified CD11c^+^ DCs isolated from TDLNs of MC38-OVA tumor-bearing *Bcl9*^f/f^*Bcl9l*^f/f^ Cre-ERT2 mice treated i.p. with tamoxifen (1 mg/100 μL) in olive oil on days -7, -6, -5, +1 and +6 post inoculation were incubated 24 hr with naive OT-1 CD8^+^ T cells at a ratio of 1:5 in an IFN-γ ELISPOT assay (*n* = 3). (**h**) Individual mice received subcutaneous injections of 2×10^6^ MC38-OVA tumor cells and treated with 40 mg/kg hsBCL9_z96_-treated MC38-OVA tumor-bearing mice every day, and 10 days after the first treatment, CD8^+^ T cells isolated from TDLNs and stimulated with 10 μg/ml of OVA-I peptide for 24 hr in an IFN-γ ELISPOT assay (*n* = 3). (**i**) *Bcl9*^f/f^*Bcl9l*^f/f^ Cre-ERT2 mice and *Bcl9*^f/f^*Bcl9l*^f/f^ mice were injected subcutaneously with 2 × 10^6^ MC38-OVA tumor cells. *Bcl9*^f/f^*Bcl9l*^f/f^ Cre-ERT2 mice and *Bcl9*^f/f^*Bcl9l*^f/f^ mice were treated i.p. with tamoxifen in olive oil on days -7, -6, -5, +1 and +6 post inoculation. At 10 days later, CD8^+^ T cells isolated from TDLNs and stimulated with 10 μg/ml of OVA-I peptide in an IFN-γ ELISPOT plate for 24 hr in an IFN-γ ELISPOT assay (*n* = 3). These data are representative values expressed as the mean ± SD of each group; *n* indicates biological replicate; Unpaired Student’s t-test.


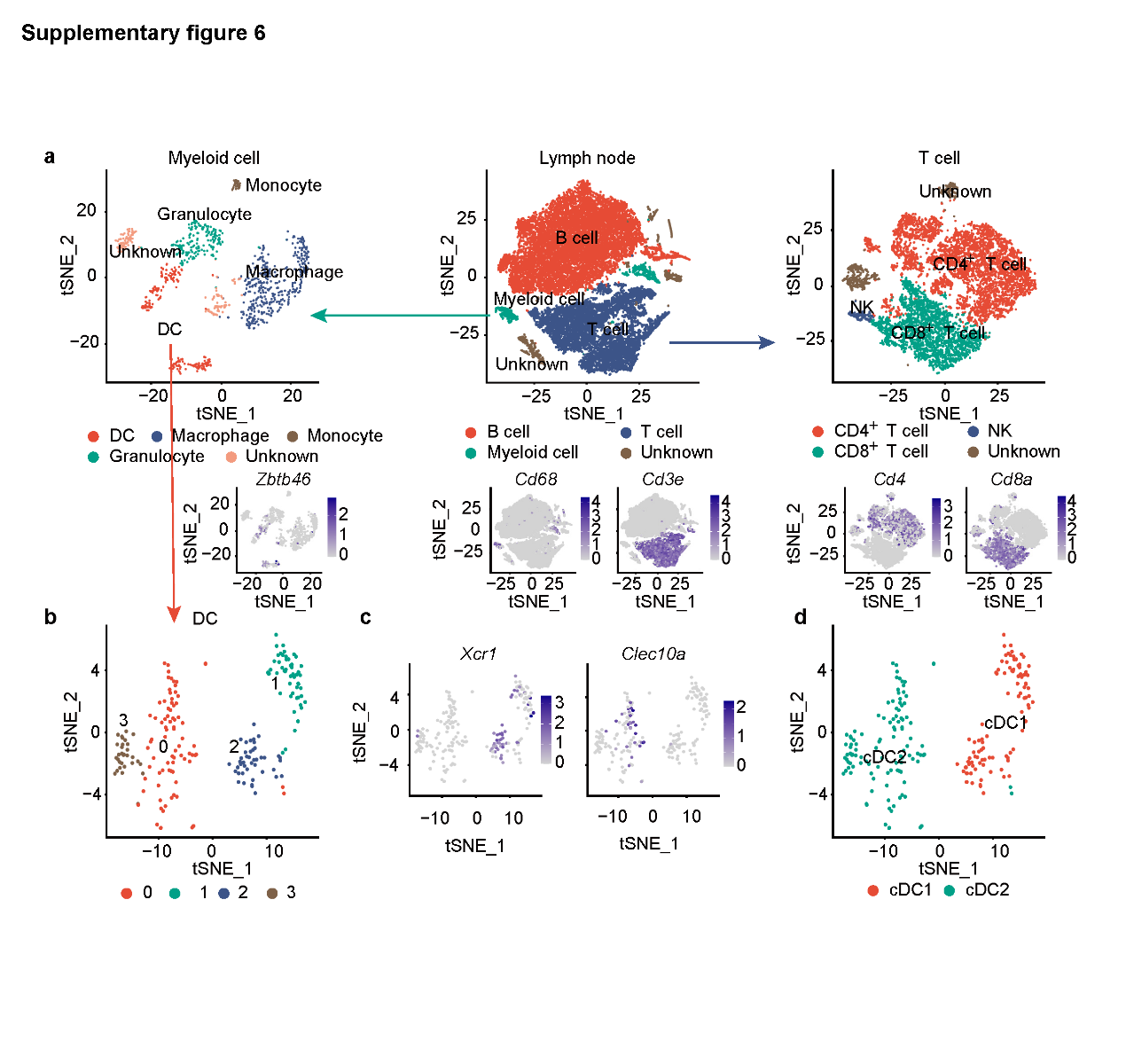


**Fig. S6. Single-cell transcriptional profiling of TdLNs from B16-OVA tumor-bearing *Bcl9/Bcl9l* deficient mice.** (**a**) The TSNE plots of clustering process and TSNE plots of marker genes (*Zbtb46* for cDCs, *Cd68* for myeloid cells, *Cd3e* for T cells, *Cd4* for CD4^+^ T cells, and *Cd8a* for CD8^+^ T cells) in TdLNs from B16-OVA tumor-bearing *Bcl9*^f/f^*Bcl9l*^f/f^ Cre-ERT2 mice and *Bcl9*^f/f^*Bcl9l*^f/f^ mice. (**b**-**d**) TSNE plots of DC reclustering (b and d) and TSNE plots of marker genes (*Xcr1* for cDC1, *Clec10a* for cDC2) (c) in TdLNs from B16-OVA tumor-bearing *Bcl9*^f/f^*Bcl9l*^f/f^ Cre-ERT2 mice and *Bcl9*^f/f^*Bcl9l*^f/f^ mice. treated i.p. with tamoxifen (1 mg/100 μL) in olive oil on days -7, -6, -5, +1, +6 and +11 post inoculation.

**
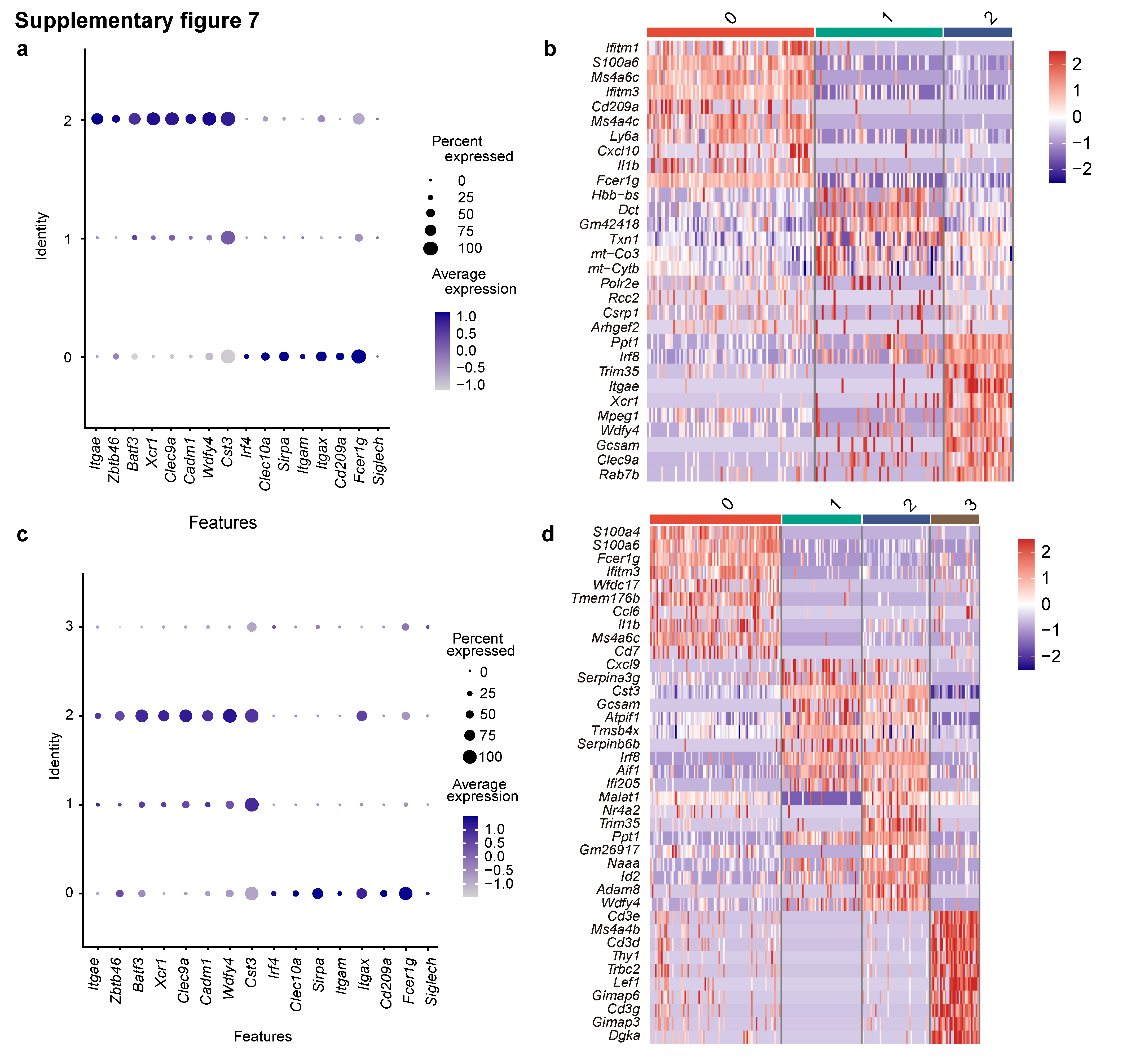
**

**Fig. S7. Single-cell transcriptional profiling of tumors and TdLNs from B16-OVA tumor-bearing *Bcl9/Bcl9l* deficient mice.** (**a**) Bubble plot of DC related genes in 3 clusters of DC in tumors from B16-OVA tumor-bearing *Bcl9*^f/f^*Bcl9l*^f/f^ Cre-ERT2 mice and *Bcl9*^f/f^*Bcl9l*^f/f^ mice treated i.p. with tamoxifen (1 mg/100 μL) in olive oil on days -7, -6, -5, +1, +6 and +11 post inoculation. (**b**) Heatmap of Top 10 differential genes in 3 clusters of DC in tumors from B16-OVA tumor-bearing *Bcl9*^f/f^*Bcl9l*^f/f^ Cre-ERT2 mice and *Bcl9*^f/f^*Bcl9l*^f/f^ mice treated i.p. with tamoxifen (1 mg/100 μL) in olive oil on days -7, -6, -5, +1, +6 and +11 post inoculation. (**c**) Bubble plot of DC related genes in 4 clusters of DC in TdLNs from B16-OVA tumor-bearing *Bcl9*^f/f^*Bcl9l*^f/f^ Cre-ERT2 mice and *Bcl9*^f/f^*Bcl9l*^f/f^ mice treated i.p. with tamoxifen (1 mg/100 μL) in olive oil on days -7, -6, -5, +1, +6 and +11 post inoculation. (**d**) Heatmap of Top 10 differential genes in 4 clusters of DC in TdLNs from B16-OVA tumor-bearing *Bcl9*^f/f^*Bcl9l*^f/f^ Cre-ERT2 mice and *Bcl9*^f/f^*Bcl9l*^f/f^ mice treated i.p. with tamoxifen (1 mg/100 μL) in olive oil on days -7, -6, -5, +1, +6 and +11 post inoculation.


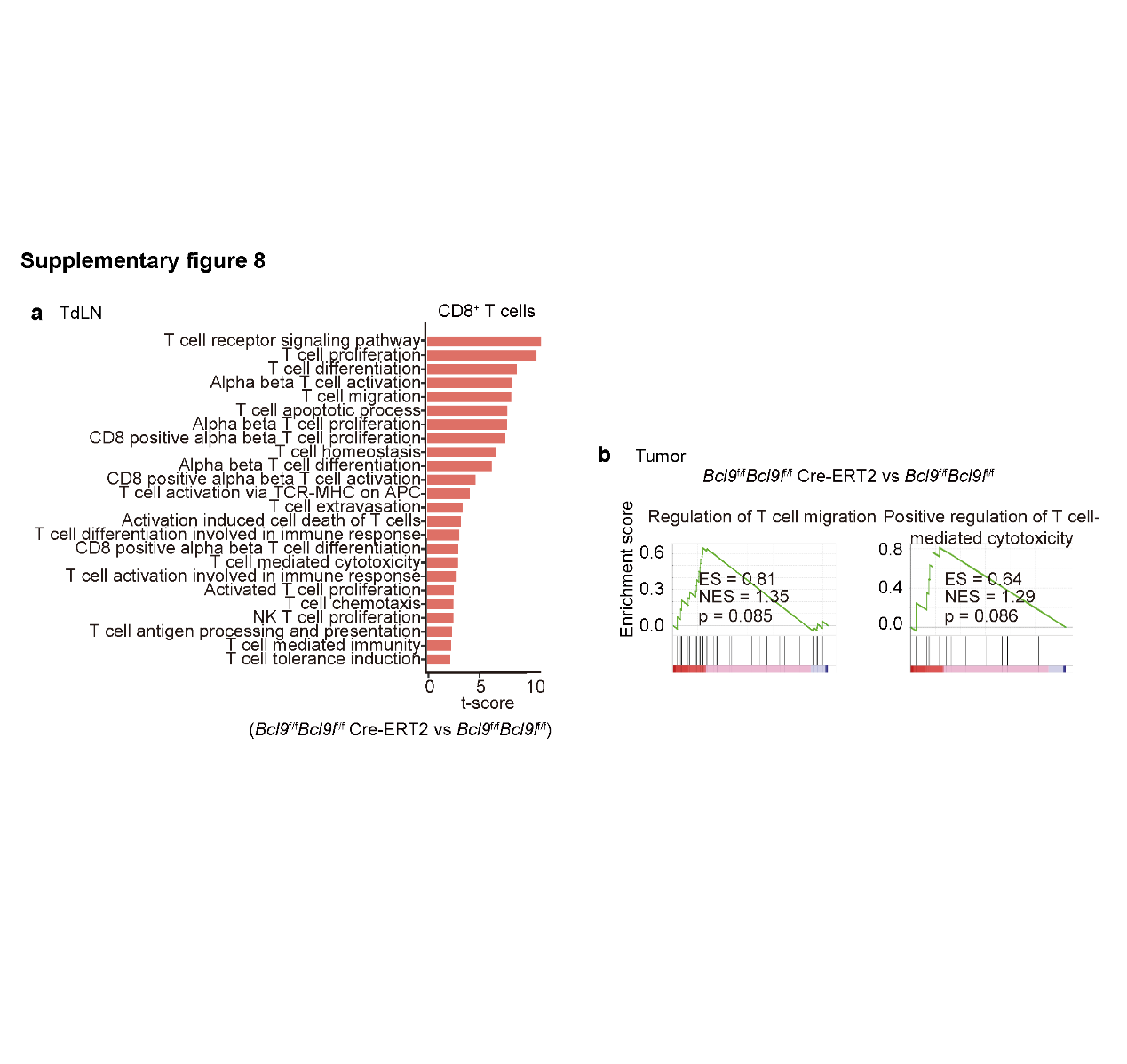


**Fig. S8. *Bcl9/Bcl9l* deficiency facilitates cDC1 activation and antigen presentation via TAK1/NF-κB/IRF1 axis using single-cell analysis.** (**a**) GSVA of CD8^+^ T cells in TdLNs from B16-OVA tumor-bearing *Bcl9*^f/f^*Bcl9l*^f/f^ Cre-ERT2 mice and *Bcl9*^f/f^*Bcl9l*^f/f^ mice treated i.p. with tamoxifen (1 mg/100 μL) in olive oil on days -7, -6, -5, +1, +6 and +11 post inoculation. The gene sets related to T cell activation and function are shown. T cell activation via TCR-MHC on APC, is the abbreviation for T cell activation via TCR contact with Ag bound to MHC on APC. (**b**) GSEA of cDC1 in tumors from B16-OVA tumor-bearing *Bcl9*^f/f^*Bcl9l*^f/f^ Cre-ERT2 mice and *Bcl9*^f/f^*Bcl9l*^f/f^ mice treated i.p. with tamoxifen (1 mg/100 μL) in olive oil on days -7, -6, -5, +1, +6 and +11 post inoculation.


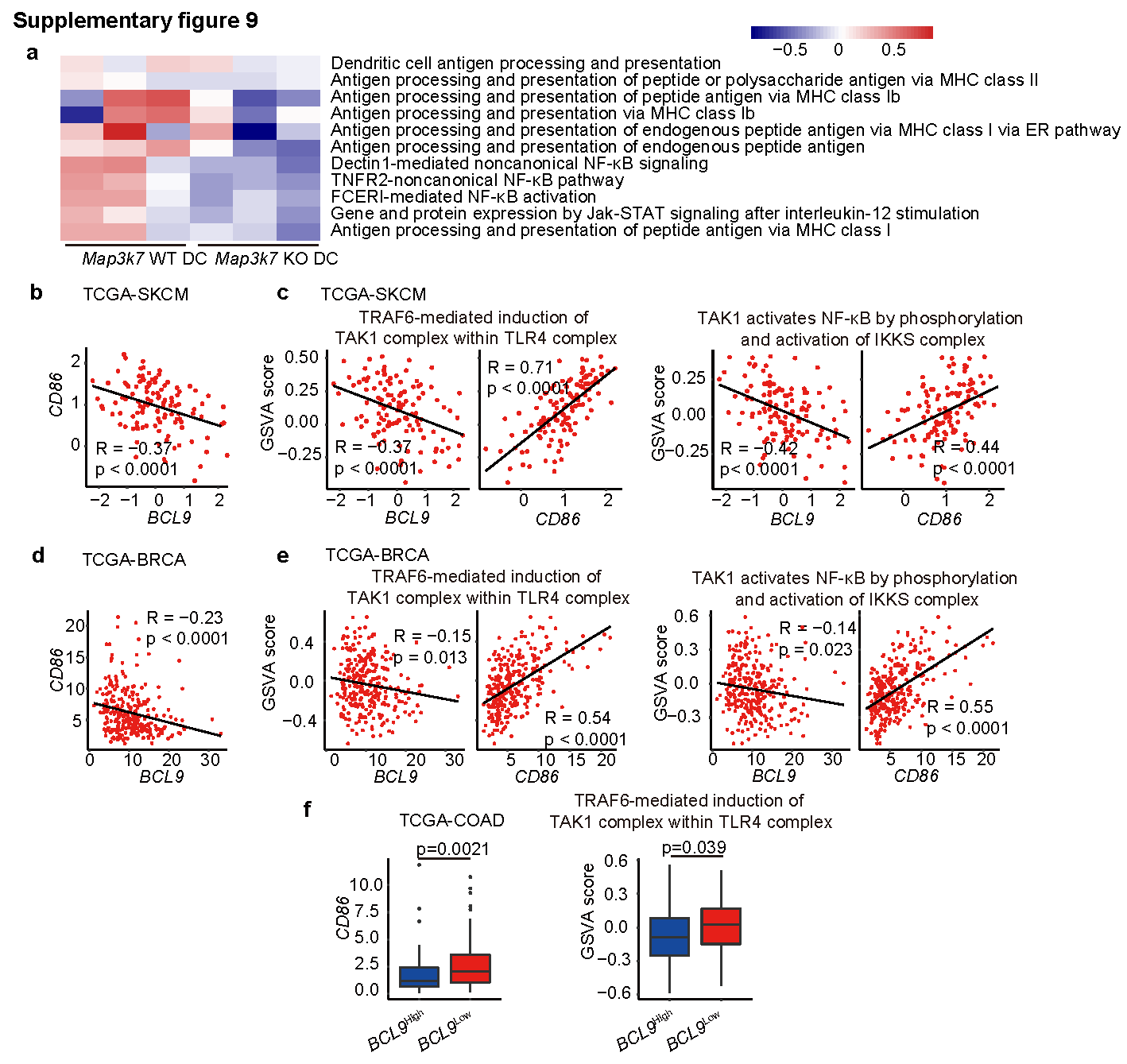


**Fig. S9. *Bcl9/Bcl9l*** **deficiency facilitates cDC1 activation and antigen presentation via TAK1/NF-κB/IRF1 axis using bioinformatics analysis.** (**a**) The heatmap showed scaled GSVA score of WT and *Map3k7* (*Tak1*) KO DC. Data was downloaded from GSE34417. (**b**) Pearson correlation between *BCL9* and *CD86* in TCGA SKCM datasets bearing high cDC1 score (Top 25%, *n* = 118). (**c**) Pearson correlation between *BCL9*, *CD86*, TLR4/TRAF6/TAK1 GSVA score and TAK1/NF-κB GSVA score in TCGA SKCM datasets bearing high cDC1 score (Top 25%, *n* = 118). (**d**) Pearson correlation between *BCL9* and *CD86* in TCGA BRCA datasets bearing high cDC1 score (Top 25%, *n* = 273). (**e**) Pearson correlation between *BCL9*, *CD86*, TLR4/TRAF6/TAK1 GSVA score and TAK1/NF-κB GSVA score in TCGA BRCA datasets bearing high cDC1 score (Top 25%, *n* = 273). (**f**) Analysis of *CD86* expression and TLR4/TRAF6/TAK1 GSVA score between low and high *BCL9* expression (top and bottom 20%) in TCGA COAD datasets (*BCL9*^Low^_,_ *n* = 91; *BCL9*^High^_,_ *n* = 91). These data are representative values are expressed as the mean ± SD of each group; *n* indicates biological replicate; Spearman’s rank-order correlation test (**b**-**e**); Unpaired Student’s t test (**f**).


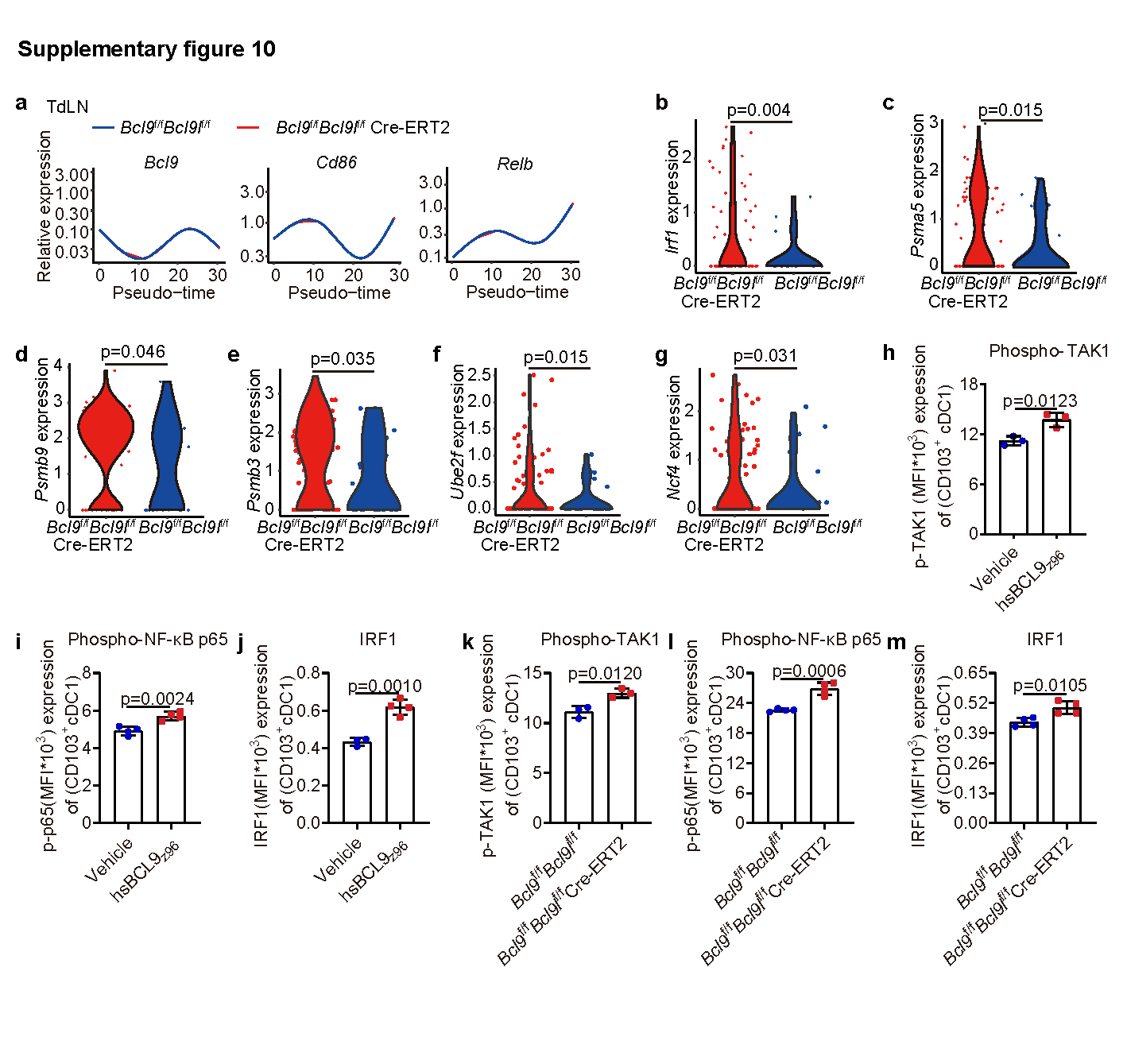
**Fig. S10. Targeting BCL9/BCL9L contributes to cDC1 activation through TAK1/NF-κB/IRF1 axis.** (**a**) Pseudo-time analysis of cDC1 in TdLNs from B16-OVA tumor-bearing *Bcl9*^f/f^*Bcl9l*^f/f^ Cre-ERT2 mice and *Bcl9*^f/f^*Bcl9l*^f/f^ mice treated i.p. with tamoxifen (1 mg/100 μL) in olive oil on days -7, -6, -5, +1, +6 and +11 post inoculation. The trend of *Bcl9*, *Cd86*, and *Relb* expression through time in TdLNs are shown. (**b**-**g**) The expression of *Irf1* (b), *Psma5* (c), *Psmb9* (d), *Psmb3* (e), *Ube2f* (f) and *Ncf4* (g) between cDC1 in tumors from B16-OVA tumor-bearing *Bcl9*^f/f^*Bcl9l*^f/f^ Cre-ERT2 mice and *Bcl9*^f/f^*Bcl9l*^f/f^ mice treated i.p. with tamoxifen (1 mg/100 μL) in olive oil on days -7, -6, -5, +1, +6 and +11 post inoculation. (**h**-**m**) Phospho-TAK1, phospho-NF-κB p65 and IRF1 expression by cDC1 of tumors from 30 mg/kg hsBCL9_z96_-treated CT26 tumor-bearing mice (h-j) and MC38 tumor-bearing *Bcl9*^f/f^*Bcl9l*^f/f^ Cre-ERT2 mice (k-m) treated i.p. with tamoxifen (1 mg/100 μL) in olive oil on days -7, -6, -5, +1, +6 and +11 post inoculation were analyzed by flow cytometry. CD103^+^ cDC1 gated on CD45^+^ CD11b^-^ CD11c^+^ MHC-II^+^ CD103^+^ cell (*n* = 3-4). These representative values denote the mean ± SD of each group derived from three independent experiments, with 'n' representing biological replicates. Statistical analysis included unpaired Student’s t tests (**b-m**).

#
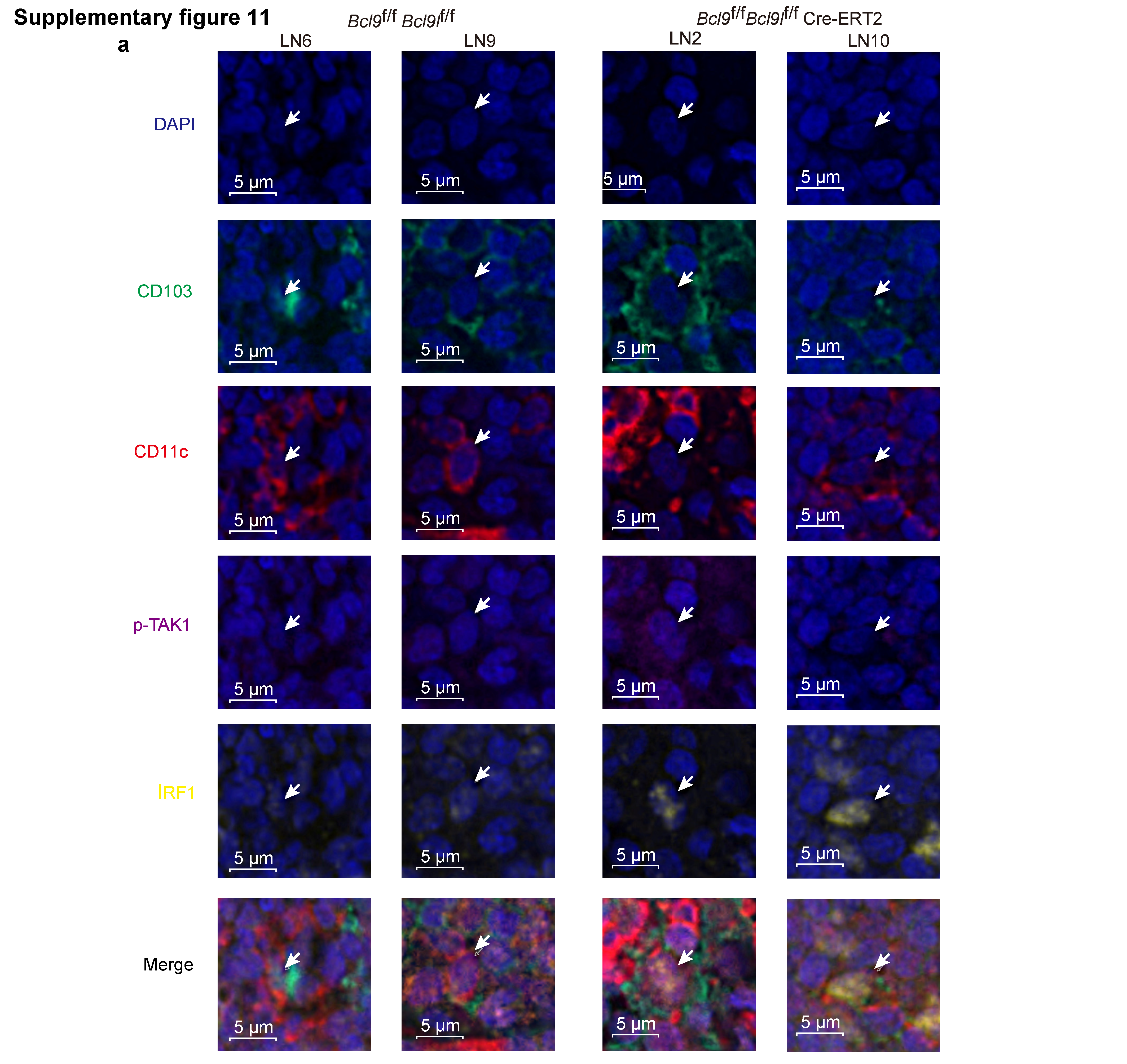


**Fig. S11. *Bcl9/Bcl9l* deficiency contributes to cDC1 activation through NF-κB/IRF1 axis.** (**a**) Representative immunofluorescence appearance of cDC1 from TdLNs of MC38 tumor-bearing *Bcl9*^f/f^*Bcl9l*^f/f^ Cre-ERT2 mice treated i.p. with tamoxifen (1 mg/100 μL) in olive oil on days -7, -6, -5, +1, +6 and +11 post inoculation were analyzed by multiplex immunofluorescence (blue for DAPI, green for CD103, red for CD11c, yellow for IRF1 and pink purple for p-TAK1). cDC1 gated on CD11c^+^ CD103^+^ cell. Scale bar, 5 μm.


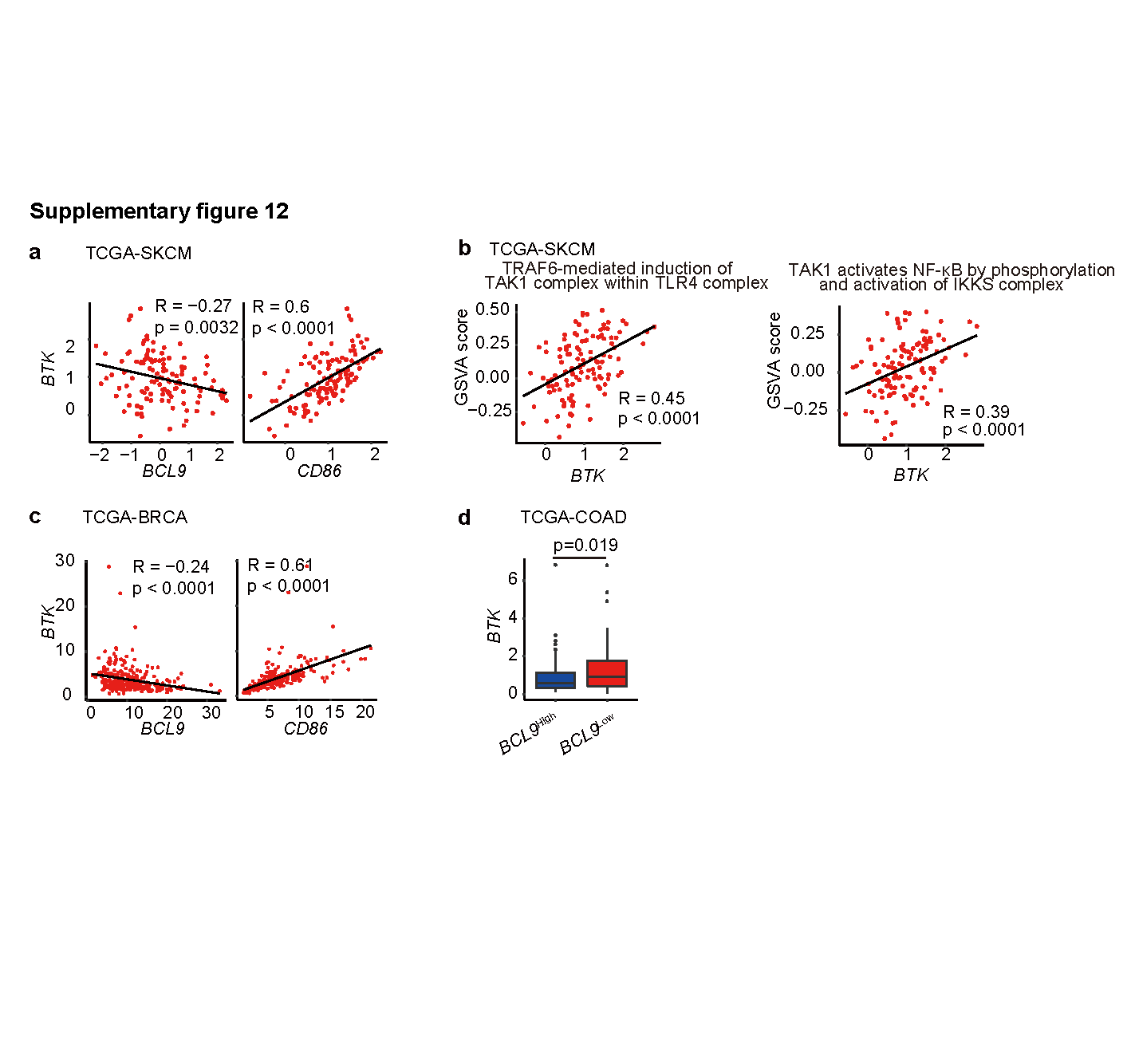


**Fig. S12. *Bcl9/Bcl9l* deficient cDC1 activate TAK1/NF-κB/IRF1 signaling by upregulating BTK expression.** (**a**) Pearson correlation between *BCL9*, *CD86*, *BTK* in TCGA SKCM datasets bearing high cDC1 score (Top 25%, *n* = 118). (**b**) Pearson correlation between *BTK*, TLR4/TRAF6/TAK1 GSVA score and TAK1/NF-κB GSVA score in TCGA SKCM datasets bearing high cDC1 score (Top 25%, *n* = 118). (**c**) Pearson correlation between *BCL9*, *CD86*, *BTK* in TCGA BRCA datasets bearing high cDC1 score (Top 25%, *n* = 273). (**d**) Analysis of *CD86* expression, TLR4/TRAF6/TAK1 GSVA score and *BTK* expression between low and high *BCL9* expression (top and bottom 20%) in TCGA COAD datasets (*BCL9*^Low^_,_ *n* = 91; *BCL9*^High^_,_ *n* = 91). These representative values denote the mean ± SD of each group derived from three independent experiments, with 'n' representing biological replicates. Statistical analysis included unpaired Student’s t tests (b-m) (**a**-**c**); Unpaired Student’s t test (**d**).


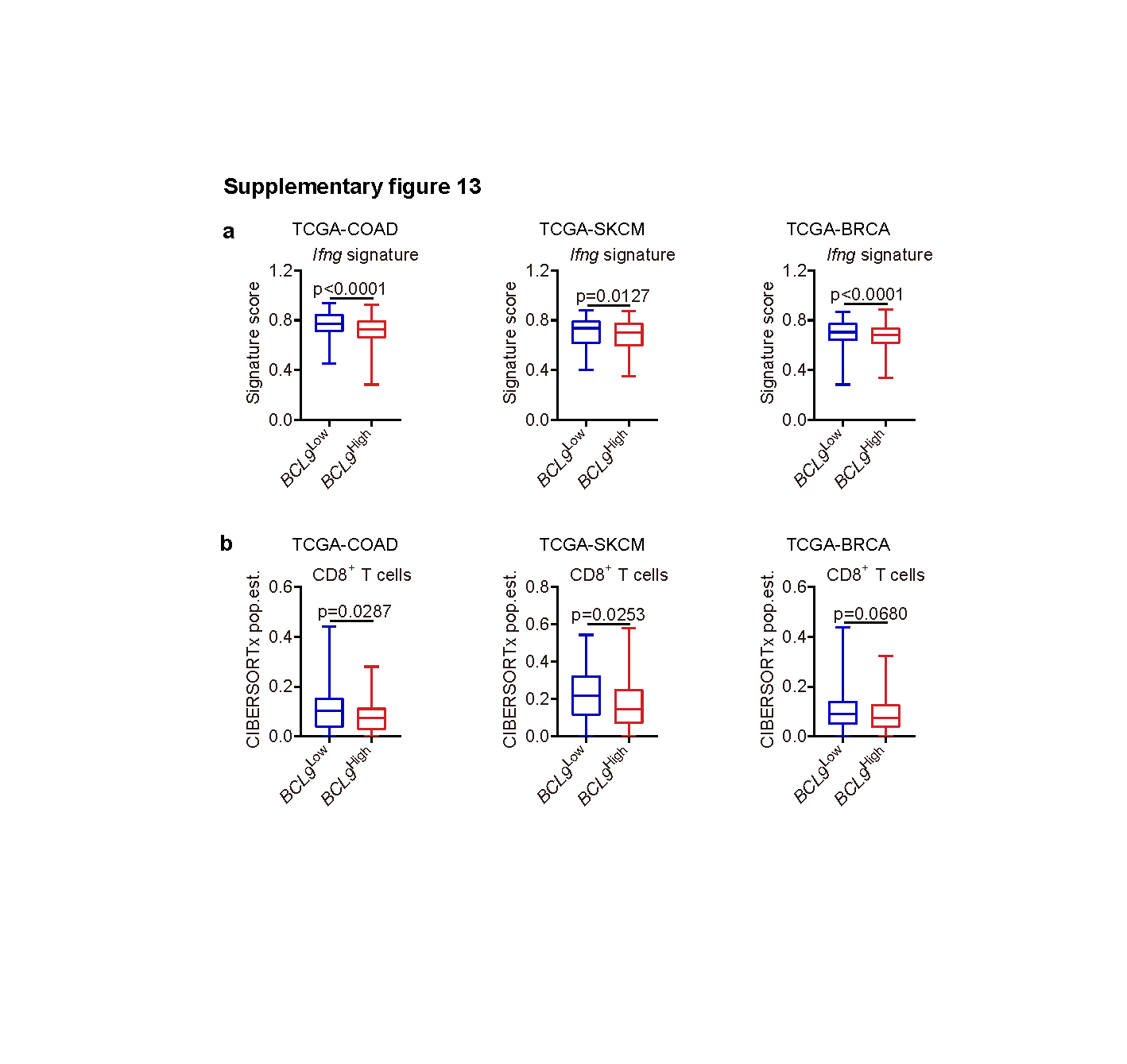
**Fig. S13. Targeting BCL9/BCL9L results in CD8^+^ T cells accumulation into tumors through CXCL9-CXCR3 axis.** (**a**) Analysis of *Ifng* signature expression between low and high *BCL9* expression (median value) based on the GSEA in TCGA datasets (TCGA COAD, *n* = 445; TCGA SKCM, *n* = 469; TCGA BRCA, *n* = 1097). (**b**) Analysis of CD8^+^ T cell infiltration between low and high *BCL9* expression (top and bottom quartile value) in TCGA datasets with Cibersort (TCGA COAD, *n* = 445; TCGA SKCM, *n* = 469; TCGA BRCA, *n* = 1097). These representative values denote the mean ± SD of each group derived from three independent experiments, with 'n' representing biological replicates. Statistical analysis included unpaired Student’s t tests (b-m)(**a** and **b**).


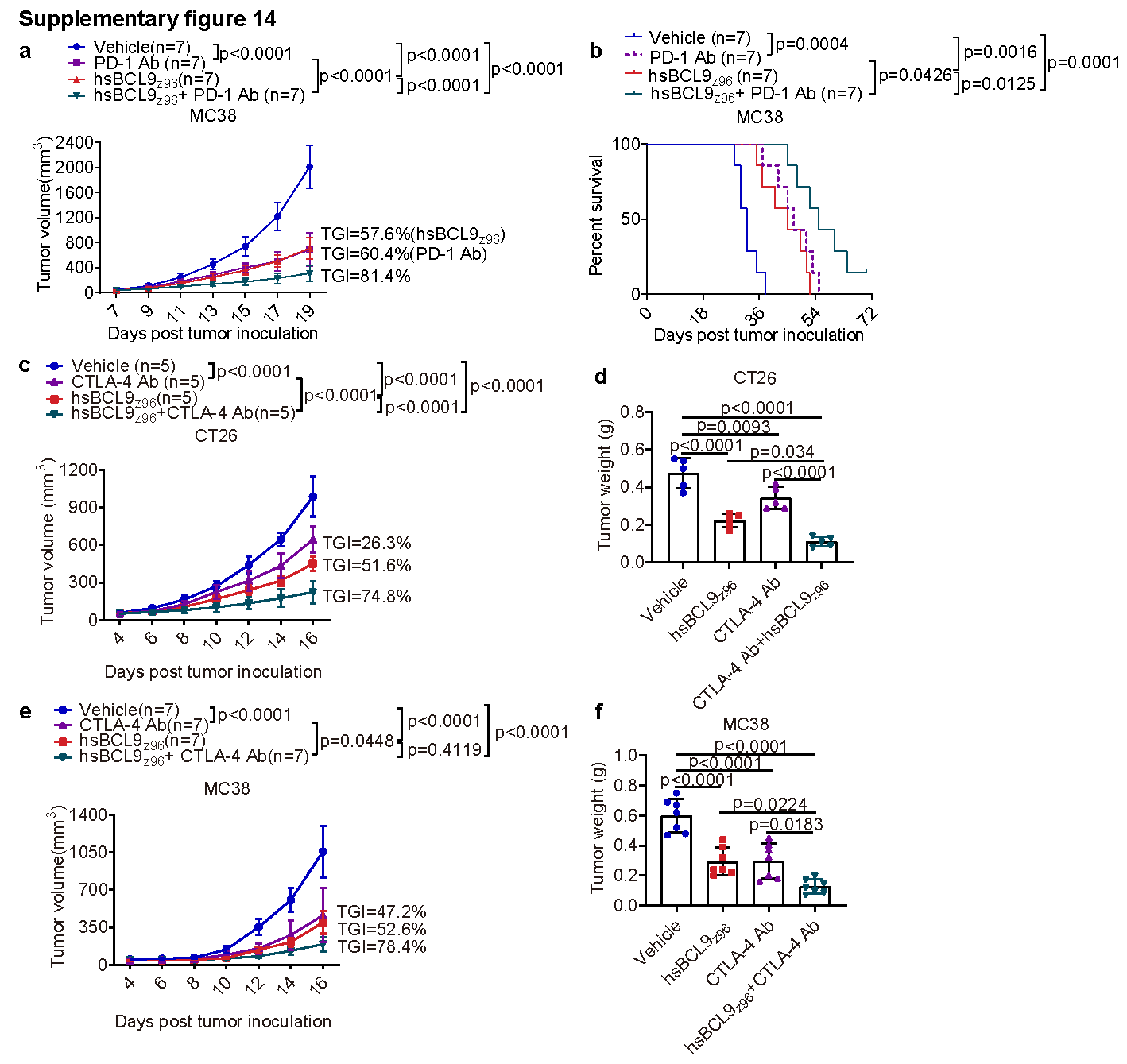
**Fig. S14. Targeting BCL9/BCL9L sensitizes tumors to immune checkpoint blockade therapy.** (**a** and **b**) Tumor growth (a) and survival (b) of MC38 tumor-bearing mice were treated i.p. with vehicle, 40 mg/kg hsBCL9_z96_ treated, 10 mg/kg anti-PD-1 or combinational therapy (*n* = 6). (**c** and **d**) Tumor growth (c) and tumor weight (d) of CT26 tumor-bearing mice were treated i.p. with vehicle, 30 mg/kg hsBCL9_z96_, 10 mg/kg anti–CTLA-4 or combinational therapy (*n* = 5). (**e** and **f**) Tumor growth (e) and tumor weight (f) of MC38 tumor-bearing mice were treated i.p. with vehicle, 40 mg/kg hsBCL9_z96_, 10 mg/kg anti-CTLA-4 or combinational therapy (*n* = 7). These representative values denote the mean ± SD of each group derived from three independent experiments, with 'n' representing biological replicate; Two-way ANOVA followed by Bonferroni test (**a**, **c** and **e**); One-way ANOVA followed by Bonferroni test (**b**, **d**, and **f**).


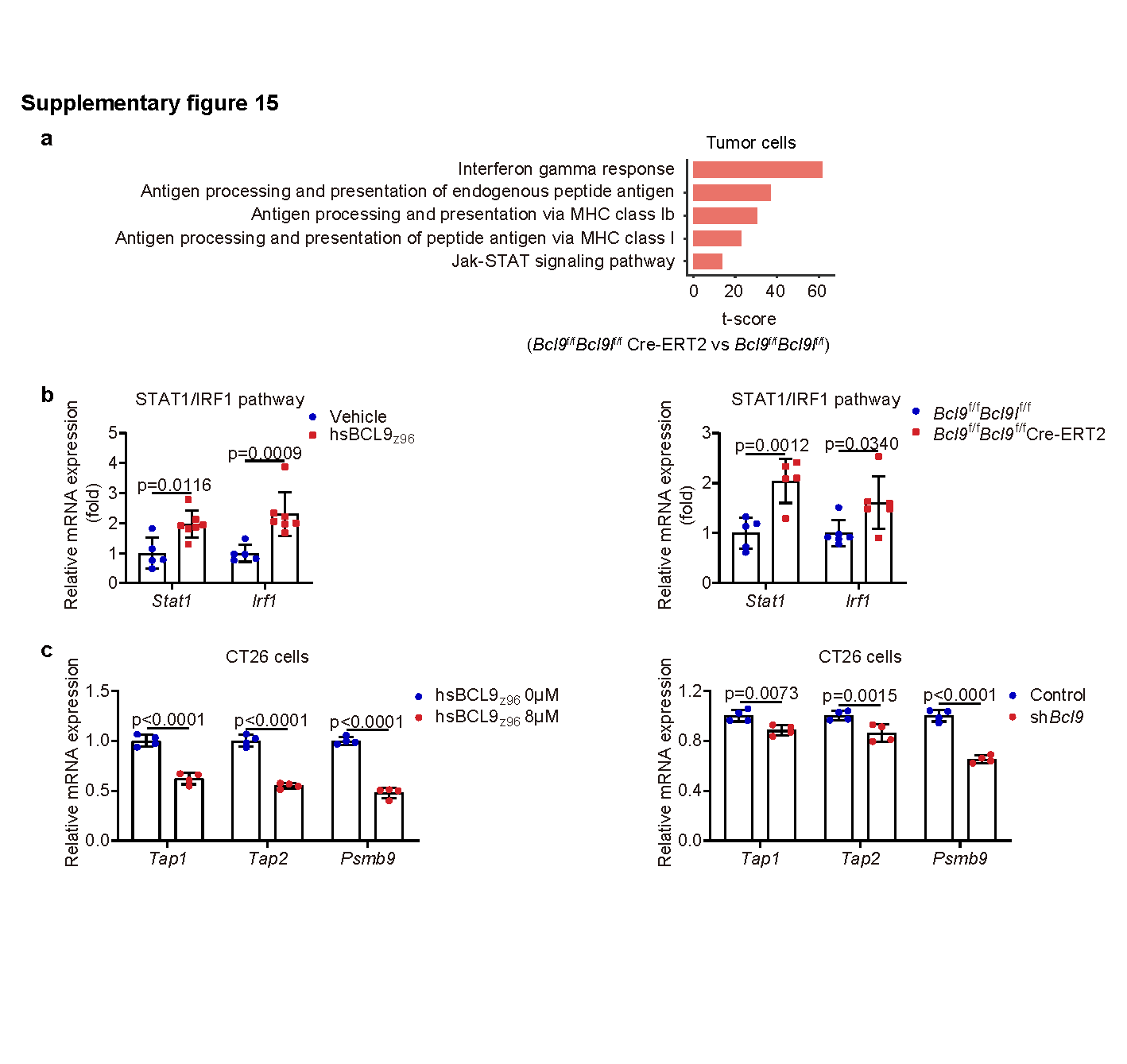


**Fig. S15. Targeting BCL9/BCL9L promotes tumor antigen presentation *in vivo*, but does not enhance antigen presentation of tumor cells *in vitro*.** (**a**) GSVA of tumor cells in tumors from B16-OVA tumor-bearing *Bcl9*^f/f^*Bcl9l*^f/f^ mice and *Bcl9*^f/f^*Bcl9l*^f/f^ Cre-ERT2 mice treated i.p. with tamoxifen (1 mg/100 μL) in olive oil on days -7, -6, -5, +1, +6 and +11 post inoculation. Antigen processing and presentation, IFN-γ response and JAK-STAT signaling pathway gene sets are shown. (**b**) The relative *Stat1* and *Irf1* mRNA expression of tumors from hsBCL9_z96_ treated CT26 tumor-bearing mice (left) and MC38 tumor-bearing *Bcl9*^f/f^*Bcl9l*^f/f^ Cre-ERT2 mice (right) treated i.p. with tamoxifen (1 mg/100 μL) in olive oil on days -7, -6, -5, +1, +6 and +11 post inoculation were analyzed by qPCR (*n* = 5-7). (**c**) The relative mRNA expression of antigen processing and presentation genes (*Tap*1, *Tap2* and *Psmb9*) in CT26 tumor cells (left) treated with the indicated doses of hsBCL9_z96_ for 24 h and in sh*Bcl9*-CT26 tumor cells (right) were analyzed by qPCR (*n* = 4-6). These representative values denote the mean ± SD of each group from three independent experiments, with 'n' indicating biological replicates. Statistical analysis involved Two-way ANOVA followed by Bonferroni test (**b** and **c**).

# Table S1. Primers for mouse identification.

| Gene | Primer |
| --- | --- |
| *Bcl9*^f/f^ F | ATGTATGAGGGGCTGGTGAG |
| *Bcl9*^f/f^ R | GCTGATGTGTGGCTAGAGCA |
| *Bcl9l*^f/f^ F | AACTGCTTGGAGAAGCATGG |
| *Bcl9l*^f/f^ R | AGGTTAACGTCCCCCAAATC |
| Cre-ERT2 F | CGTGATCTGCAACTCCAGTC |
| Cre-ERT2 *R* | AGGCAAATTTTGGTGTACGG |
| Cre-ERT2 WT F | CTGGCTTCTGAGGACCG |
| Cre-ERT2 WT R | CCGAAAATCTGTGGGAAGTC |
| *TCRα* F | CAGCAGCAGGTGAGACAAAGT |
| *TCRα* R | GGCTTTATAATTAGCTTGGTCC |

# Table S2. Primers for real-time PCR.

| Gene | Primer |
| --- | --- |
| m*Actb* F | CGTTGACATCCGTAAAGACC |
| m*Actb* R | AACAGTCCGCCTAGAAGCAC |
| m*Cxcl9* F | GGAGTTCGAGGAACCCTAGTG |
| m*Cxcl9* R | GGGATTTGTAGTGGATCGTGC |
| m*Ifng* F | ATGAACGCTACACACTGCATC |
| m*Ifng* R | CCATCCTTTTGCCAGTTCCTC |
| m*Xcl1* F | TAGCTGTGTGAACTTACAAACCC |
| m*Xcl1* R | ACAGTCTTGATCGCTGCTTTC |
| m*Tap1* F | GGACTTGCCTTGTTCCGAGAG |
| m*Tap1* R | GCTGCCACATAACTGATAGCGA |
| m*Tap2* F | CTGGCGGACATGGCTTTACTT |
| m*Tap2* R | CTCCCACTTTTAGCAGTCCCC |
| m*B2m* F | TTCTGGTGCTTGTCTCACTGA |
| m*B2m* R | CAGTATGTTCGGCTTCCCATTC |
| m*Psmb9* F | CATGAACCGAGATGGCTCTAGT |
| m*Psmb9* R | TCATCGTAGAATTTTGGCAGCTC |
| m*Stat1* F | TCACAGTGGTTCGAGCTTCAG |
| m*Stat1* R | GCAAACGAGACATCATAGGCA |
| m*Irf1* F | ATGCCAATCACTCGAATGCG |
| m*Irf1* R | TTGTATCGGCCTGTGTGAATG |

# Table S3. Antibodies for flow cytometry.

| Antibody | Source | Identifier |
| --- | --- | --- |
| Anti-mouse CD45 PerCP-Cyanine5.5 | eBioscience | Cat:45-0451-82 |
| Anti-mouse CD45 APC/Cyanine7 | BioLegend | Cat:157617 |
| Anti-mouse CD3e FITC | eBioscience | Cat:11-0031-82 |
| Anti-mouse CD3e PerCP/Cyanine5.5 | BioLegend | Cat:100327 |
| Anti-mouse CD3e eFluor450 | eBioscience | Cat:48-0031-80 |
| Anti-mouse CD8a APC | eBioscience | Cat:17-0088-42 |
| Anti-mouse CD8a APC-eFluor780 | eBioscience | Cat:47-0081-82 |
| Anti-mouse CD8a FITC | eBioscience | Cat:11-0081-82 |
| Anti-mouse CD103 PE | eBioscience | Cat:12-1031-82 |
| Anti-mouse CD103 PerCP/Cyanine5.5 | BioLegend | Cat:121415 |
| Anti-mouse CD11c eFluor450 | eBioscience | Cat:48-0114-82 |
| Anti-mouse CD11c PE-eFluor610 | eBioscience | Cat:61-0114-80 |
| Anti-mouse CD11c FITC | BioLegend | Cat:117305 |
| Anti-mouse CD11b APC | eBioscience | Cat:17-0112-81 |
| Anti-mouse CD11b PE-Cyanine7 | eBioscience | Cat:25-0118-41 |
| Anti-mouse CXCL9 eFluor660 | eBioscience | Cat:50-3009-80 |
| Anti-mouse XCR1 APC | BioLegend | Cat:148205 |
| Anti-mouse MHC-II eFluor450 | eBioscience | Cat:48-5321-82 |
| Anti-mouse MHC-II FITC | eBioscience | Cat:11-5321-81 |
| Anti-mouse CD80 FITC | BioLegend | Cat:104705 |
| Anti-mouse CD86 APC | BioLegend | Cat:105011 |
| Anti-mouse CD86 PE | BioLegend | Cat:159203 |
| Anti-mouse CD40 PE | eBioscience | Cat:12-0401-81 |
| Anti-mouse Granzyme B eFluor450 | eBioscience | Cat:48-8898-80 |
| Anti-mouse Ki67 APC | eBioscience | Cat:17-5698-80 |
| Anti-mouse IFN-γ PE | eBioscience | Cat:12-7311-81 |
| Anti-mouse NK1.1 PE-Cyanine7 | eBioscience | Cat:25-5941-81 |
| MHC I-Strept H-2 K^b^ OVA SIINFEKL | iBa | Cat:6-7015-001 |
| Strept-Tactin APC for MHC I Streptamers | iBa | Cat:6-5010-001 |
| Anti-mouse XCL1/lymphotactin | Sino Biological | Cat:50677-R002 |
| Anti- mouse IRF-1 (D5E4) XP | Cell Signaling Technology | Cat:8478 |
| Anti-mouse-phospho-NF-κB p65 (Ser536) | Cell Signaling Technology | Cat:3033 |
| Anti-mouse/human p-TAK1 Phospho-TAK1(Thr184 + Thr187) | Bioss | Cat:bs-3439R |
| Goat anti-rabbit IgG H&L (PE) | abcam | Cat:ab72465 |

# Reference

1 Feng, M. et al. Pharmacological inhibition of β-catenin/BCL9 interaction overcomes resistance to immune checkpoint blockades by modulating T(reg) cells. Science advances 5, eaau5240, doi:10.1126/sciadv.aau5240 (2019).

2 Liu, Y. et al. Comparative Molecular Analysis of Gastrointestinal Adenocarcinomas. Cancer cell 33, 721-735.e728, doi:10.1016/j.ccell.2018.03.010 (2018).

3 Zeng, D. et al. IOBR: Multi-Omics Immuno-Oncology Biological Research to Decode Tumor Microenvironment and Signatures. Frontiers in immunology 12, 687975, doi:10.3389/fimmu.2021.687975 (2021).

4 Lin, A., Zhang, J. & Luo, P. Crosstalk Between the MSI Status and Tumor Microenvironment in Colorectal Cancer. Frontiers in immunology 11, 2039, doi:10.3389/fimmu.2020.02039 (2020).

5 Ayers, M. et al. IFN-γ-related mRNA profile predicts clinical response to PD-1 blockade. The Journal of clinical investigation 127, 2930-2940, doi:10.1172/jci91190 (2017).

6 Mariathasan, S. et al. TGFβ attenuates tumour response to PD-L1 blockade by contributing to exclusion of T cells. Nature 554, 544-548, doi:10.1038/nature25501 (2018).

7 Liberzon, A. et al. Molecular signatures database (MSigDB) 3.0. Bioinformatics (Oxford, England) 27, 1739-1740, doi:10.1093/bioinformatics/btr260 (2011).

8 Dixon, K. O. et al. TIM-3 restrains anti-tumour immunity by regulating inflammasome activation. Nature 595, 101-106, doi:10.1038/s41586-021-03626-9 (2021).

9 Yoshihara, K. et al. Inferring tumour purity and stromal and immune cell admixture from expression data. Nature communications 4, 2612, doi:10.1038/ncomms3612 (2013).

10 Sanchez-Vega, F. et al. Oncogenic Signaling Pathways in The Cancer Genome Atlas. Cell 173, 321-337.e310, doi:10.1016/j.cell.2018.03.035 (2018).

11 Newman, A. M. et al. Robust enumeration of cell subsets from tissue expression profiles. Nature methods 12, 453-457, doi:10.1038/nmeth.3337 (2015).

12 Hänzelmann, S., Castelo, R. & Guinney, J. GSVA: gene set variation analysis for microarray and RNA-seq data. BMC bioinformatics 14, 7, doi:10.1186/1471-2105-14-7 (2013).

13 Subramanian, A. et al. Gene set enrichment analysis: a knowledge-based approach for interpreting genome-wide expression profiles. Proceedings of the National Academy of Sciences of the United States of America 102, 15545-15550, doi:10.1073/pnas.0506580102 (2005).

14 Efremova, M., Vento-Tormo, M., Teichmann, S. A. & Vento-Tormo, R. CellPhoneDB: inferring cell-cell communication from combined expression of multi-subunit ligand-receptor complexes. Nature protocols 15, 1484-1506, doi:10.1038/s41596-020-0292-x (2020).

15 Aibar, S. et al. SCENIC: single-cell regulatory network inference and clustering. Nature methods 14, 1083-1086, doi:10.1038/nmeth.4463 (2017).

16 Trapnell, C. et al. The dynamics and regulators of cell fate decisions are revealed by pseudotemporal ordering of single cells. Nature biotechnology 32, 381-386, doi:10.1038/nbt.2859 (2014).
